# Interplay Between ncRNAs and Cellular Communication: A Proposal for Understanding Cell-Specific Signaling Pathways

**DOI:** 10.3389/fgene.2019.00281

**Published:** 2019-04-02

**Authors:** Santiago Ramón y Cajal, Miguel F. Segura, Stefan Hümmer

**Affiliations:** ^1^Department of Pathology, Vall d’Hebron University Hospital, Universitat Autònoma de Barcelona, Barcelona, Spain; ^2^Translational Molecular Pathology, Vall d’Hebron Research Institute, Barcelona, Spain; ^3^Spanish Biomedical Research Network Centre in Oncology (CIBERONC), Barcelona, Spain; ^4^Group of Translational Research in Child and Adolescent Cancer, Vall d’Hebron Research Institute, Barcelona, Spain

**Keywords:** cancer, cellular communication, cell signaling, long non-coding RNA, microRNA, epigenetics

## Abstract

Intercellular communication is essential for the development of specialized cells, tissues, and organs and is critical in a variety of diseases including cancer. Current knowledge states that different cell types communicate by ligand–receptor interactions: hormones, growth factors, and cytokines are released into the extracellular space and act on receptors, which are often expressed in a cell-type-specific manner. Non-coding RNAs (ncRNAs) are emerging as newly identified communicating factors in both physiological and pathological states. This class of RNA encompasses microRNAs (miRNAs, well-studied post-transcriptional regulators of gene expression), long non-coding RNAs (lncRNAs) and other ncRNAs. lncRNAs are diverse in length, sequence, and structure (linear or circular), and their functions are described as transcriptional regulation, induction of epigenetic changes and even direct regulation of protein activity. They have also been reported to act as miRNA sponges, interacting with miRNA and modulating its availability to endogenous mRNA targets. Importantly, lncRNAs may have a cell-type-specific expression pattern. In this paper, we propose that lncRNA–miRNA interactions, analogous to receptor–ligand interactions, are responsible for cell-type-specific outcomes. Specific binding of miRNAs to lncRNAs may drive cell-type-specific signaling cascades and modulate biochemical feedback loops that ultimately determine cell identity and response to stress factors.

## Introduction

Cancer is a complex disease and a major cause of death worldwide. Development of neoplastic disease is a multistep process involving the accumulation of numerous molecular changes. These changes impact cellular function within the tumor and its microenvironment, ultimately resulting in the hallmarks of cancer (Hanahan and Weinberg, 2011).

To date, most researchers have aimed to define the molecular mechanisms of tumorigenesis and cancer progression based on the classical gene expression theory – transcription of coding genes followed by protein synthesis. Following this approach, numerous genetic (e.g., mutations and genomic aberrations) and epigenetic alterations have been identified to have an association with carcinogenesis (Lengauer et al., 1998; Kagohara et al., 2018). In addition, alterations in post-transcriptional regulation of gene expression (e.g., splicing), mRNA translation (e.g., miRNAs) and post-translational protein modification (e.g., phosphorylation) have been reported in almost every type of cancer. When different combinations are taken into consideration, the potential alterations are almost infinite. However, studies have mainly been based on around 20,000 protein-coding genes, corresponding to approximately 2% of the whole transcribed genome (Bertone et al., 2004; Carninci et al., 2005); the other transcripts include a large variety of non-coding RNAs (ncRNAs). Continuous generation of RNA sequencing (RNAseq) data shows that ncRNAs are strongly deregulated in pathological processes – particularly in multifactorial diseases like cancer (Cipolla et al., 2018). Hence, current limitations to deciphering the molecular mechanisms of cancer might be due to the fact that the putative implications of a large part of the genome remain undefined.

While ncRNA genes were for years considered as an irrelevant part the genome there is growing evidence that mammalian cells produce them in their thousands (Bertone et al., 2004; Carninci et al., 2005). Yet, in the absence of experimental verification of their function, most (>95%) of these transcripts are still considered transcriptional noise (Ponjavic et al., 2007). Studies dating back to the early 1990s indicated that certain lncRNAs may have similar functions to common mRNAs (Brannan et al., 1990; Brown et al., 1991), and since then, detailed studies on certain well-characterized non-coding transcripts have provided mechanistic insights. However, the variety in their mode of action, ranging from protein activity regulation to epigenetic control and regulation of other ncRNAs, implies that we are just beginning to understand their importance for a multitude of biochemical and cellular functions. Importantly, the role of these transcripts in certain tumor types is beginning to become apparent, and lncRNA expression profile has been proposed as a strong prognostic factor (Jiang et al., 2016; Bolha et al., 2017; Ali et al., 2018). It is therefore conceivable that elucidating the function of lncRNAs in normal cells and their deregulation in cancer cells will be one of the next milestones toward a more detailed understanding of the molecular mechanisms of cancer.

In this review, we propose a new role of ncRNAs in cancer. This model, analogous to the well-established ligand–receptor interactions, proposes intercellular communication via ncRNA interactions as a fundamental concept in cancer. This model could provide a hypothetical basis to explain the different types of biochemical feedback in tissues, which in turn could be linked to the differing response to drugs in tumors harboring similar genetic alterations, the different sites of tumor metastases, and the activation of different microRNA profiles depending on the tumor type and location.

## The Non-Coding Transcriptome

RNAs comprise a diverse range of molecules. In addition to the well-characterized RNAs with established functions such as coding genes (mRNAs), protein synthesis (rRNAs and tRNAs), or mRNA splicing (snRNAs), a multitude of additional non-protein-coding RNAs has been described in recent years. According to their size, ncRNAs are categorized as small (<200 bp) or long non-coding RNAs (lncRNAs, >200 bp).

## Small Non-Coding RNAS

The group of short ncRNAs consists of microRNAs (miRNAs), small interference RNAs (siRNAs), small nucleolar (snoRNAs), and Piwi-interacting RNAs (piRNAs) (Zamore and Haley, 2005). Of these, miRNAs have been best characterized in terms of their function, regulation and role in multiple human diseases such as cancer. Initiated by the discovery of the first miRNA, lin-4 in *Caenorhabditis elegans* (Lee et al., 1993; Wightman et al., 1993), large research efforts have led to a detailed characterization of miRNA biogenesis and regulatory functions in recent years (Ambros, 2004; Bartel, 2004; He and Hannon, 2004; Ebert and Sharp, 2012). Binding of miRNAs to specific sites in their target transcripts, called miRNA recognition elements (MRE), results in either transcript degradation or translational inhibition (Lee et al., 1993; Shivdasani, 2006). Currently there are just under 2,000 human high-confidence annotated miRNAs (Kozomara et al., 2018), and it is believed that they collectively regulate at least one third of the genome (Hammond, 2015). Importantly, gene expression profiling studies have demonstrated altered miRNA expression in a wide range of human diseases, including cancer.

## miRNAs and Cancer

miRNAs have been shown to participate in cancer throughout the various stages: from tumor origin, to immortalization, metastatic steps and interactions with the host tissue (Saliminejad et al., 2018), and they are able to regulate oncogenes and tumor suppressor genes. In the clinical setting, miRNA expression signatures are emerging as important diagnostic and prognostic predictors (Cho, 2007; Kong et al., 2012; Hayes et al., 2014; Drusco and Croce, 2017). Functional studies clearly support a relevant role of certain miRNAs in cancer. However, a remaining challenge is to understand the exact signaling pathways altered by miRNA deregulation. Several factors contribute to this complexity: (1) the 3′ UTR of a particular target gene contains multiple MREs; (2) multiple MREs can act either alone or cooperatively and (3) the same miRNA can regulate different targets (Doench and Sharp, 2004; Grimson et al., 2007).

In addition to cell-type-specific gene expression profiles, deregulation of miRNAs can result in tumor suppressive or oncogenic effects in a context-dependent manner (Zhang et al., 2007). Furthermore, the existence of feedback loops involving certain transcription factors such as c-Myc, which is both a regulator of miRNA expression and a target of miRNAs, adds yet another layer of complexity to the role of miRNAs in cancer (Jackstadt and Hermeking, 2015).

## miRNA Trafficking

While the main mechanism of action is to control mRNA stability or translation in the cytoplasm, miRNAs can be found in unexpected cellular compartments such as the nucleus, mitochondria, and endoplasmic reticulum (Leung, 2015). They are also found in the extracellular space – this was first described in 2008, when it was proposed that circulating miRNAs may serve as biomarkers of certain cancers (Mitchell et al., 2008). Since then, miRNAs have been discovered in various extracellular environments including blood (Weber et al., 2010), urine (Gidlof et al., 2011), saliva (Park et al., 2009; Patel et al., 2011), and ascitic fluid (Husted et al., 2011). These findings opened up two new fields of investigation on miRNA. First, as they are easy to detect in body fluids, clinical research has focused on the use of extracellular miRNAs as biomarkers as an alternative, non-invasive method for diagnosis and disease monitoring (Duttagupta et al., 2011; Ajit, 2012). Second, because miRNAs are found in the extracellular space, it was proposed that they may act not only in the cells in which they are transcribed but also in neighboring cells. This intriguing idea of horizontal transfer of genetic material subsequently gained major attention. To date, five non-exclusive mechanisms of miRNA release from donor cells have been proposed: (i) miRNA bound to RNA-protein complexes (e.g., in complex with Argonaut) (Arroyo et al., 2011; Turchinovich et al., 2011); (ii) transport via lipid or lipoprotein particles (Rayner and Hennessy, 2013); (iii) vesicles shed directly from the plasma membrane (Hunter et al., 2008; Callis et al., 2009; Shefler et al., 2010; Jaiswal et al., 2012); (iv) vesicles of endosomal origin (exosomes) (Valadi et al., 2007; Skog et al., 2008; Rechavi et al., 2009) and (v) vesicles from apoptotic bodies (Zernecke et al., 2009; Hergenreider et al., 2012). Currently, the coexistence of these different forms of miRNA transport is supported in the literature, but improved biochemical methods and molecular tools with higher temporal and spatial resolution are required to strengthen the evidence (Tkach and Thery, 2016).

Many groups have clearly demonstrated that isolated miRNA containing fractions (e.g., exosomes) from donor cells are capable of inducing phenotypic alterations in the recipient cells. However, studies on the underlying mechanisms are rare thus far, and basic questions concerning the amount of miRNA (signaling-like or enzymatic function) or the type of miRNA [individual or pool of miRNA(s)] remain unaddressed.

## Long Non-Coding RNAs

All RNAs longer than 200 nucleotides that are not translated into proteins are collectively categorized as lncRNAs (Carninci et al., 2005; Guttman et al., 2009; Ponting et al., 2009; Cabili et al., 2011; Derrien et al., 2012; Housman and Ulitsky, 2016; Lagarde et al., 2017). Similarly to mRNAs, lncRNAs are transcribed by RNA polymerase II and are often subject to post-transcriptional modification like 5′ capping, 3′ poly-adenylation and splicing (Quinn and Chang, 2016). Although less well-studied than miRNAs, several thousand lncRNAs have already been described, and thanks to technical advances in RNAseq techniques and computational prediction methods, the total number of lncRNAs identified continues to increase (Quinn and Chang, 2016; Hon et al., 2017). Solely defined by their length, lncRNAs constitute the largest class of ncRNAs in the mammalian genome, and can be further categorized into long intergenic ncRNAs (lincRNAs), antisense RNAs (asRNAs), pseudogenes, and circular RNAs (circRNAs) (Quinn and Chang, 2016; Lagarde et al., 2017). lncRNAs fulfill a variety of functions by interacting with DNA, RNA and proteins, and they may be described according to their mechanism of action toward their interacting molecule as enhancers, decoys, guides or scaffolds (Wang and Chang, 2011; Fok et al., 2017; Kopp and Mendell, 2018).

The first unsupervised clustering analysis of individual transcripts in different tissues revealed that 78% of lncRNAs (in comparison to 19% of mRNAs) were expressed in a tissue-specific manner (Cabili et al., 2011). As sequencing techniques advanced, this specificity of expression has been observed at the individual cell level, and even differential cell-to-cell expression has been observed by Lv et al. (2016); we could confirm this in our own (unpublished) studies in the triple negative breast cancer cell line MB-MDA231. In the field of cancer biology, lncRNA expression profile has recently been proposed as a strong prognostic factor and even as a therapeutic target (Chen et al., 2014; Yarmishyn and Kurochkin, 2015).

Despite the growing catalog of lncRNAs, the majority of detected transcription products remain functionally unannotated. Gene function prediction based on sequence homology for protein-coding genes is challenging (Ulitsky, 2016). Therefore, lncRNA classification requires either specialized computational tools or genome-wide functional studies as currently performed by the use of gene knockout techniques (e.g., CRISPERi) (Gilbert et al., 2013; Goyal et al., 2017).

## lncRNAs in Cancer

Differential expression of lncRNAs has been described in a variety of pathological conditions including cardiovascular, autoimmune, neurodegenerative diseases and particularly in cancer (Walsh et al., 2014; Nguyen and Carninci, 2016; Zhang et al., 2016; Aune et al., 2017; Bhan et al., 2017; Viereck and Thum, 2017; Wan et al., 2017). The first reported lncRNA with an aberrant expression in cancer was prostate cancer associated 3 (PCA3) (Bussemakers et al., 1999), which was identified via differential display analysis of transcripts in normal human prostate cancer. PCA3 was the first FDA-approved lncRNA-based biomarker for use in clinical practice. Since then, it has proven a useful, non-invasive test for prostate cancer (Wei et al., 2014). In subsequent years, many other lncRNAs have been identified as having a highly predictive value in the diagnosis of different cancers. Among those, deregulated expression of the lncRNA HOTAIR, originally described in breast cancer, is associated with cancer progression in 26 human tumor types (Gupta et al., 2010; Bhan and Mandal, 2015; Teschendorff et al., 2015). Recently, the application of next-generation sequencing in a variety of different cancer transcriptomes uncovered thousands of lncRNAs with aberrant expression in different cancer types (Huarte, 2015). These numbers increase further when single nucleotide polymorphisms (SNPs) that are associated with cancer are taken into account (Freedman et al., 2011).

Regarding metastasis, some lncRNAs have been associated with more aggressive, metastatic tumors and even with cancer cell colonization to specific organ sites (Li et al., 2018). For example, in colorectal carcinoma, expression of the lncRNA CCAT2 correlated with a higher incidence of liver metastasis (Ling et al., 2013). Another example is the association of elevated levels of HOTAIR with a higher incidence of liver metastasis in gastric cancer (Zhang et al., 2015) and with brain metastasis in non-small cell lung carcinoma (Nakagawa et al., 2013).

Despite the enormous number of lncRNAs described as having aberrant expression in different cancer types, to date, only a few have been functionally characterized (reviewed in Huarte, 2015); existing studies have identified tumor-suppressor and oncogenic functions of lncRNAs (Prensner and Chinnaiyan, 2011; Huarte, 2015). In many cases, the functional role of these lncRNAs has been linked to well-known oncogenic pathways like p53 and c-myc or participating in different steps of classical cancer processes such as epithelial mesenchymal transition (EMT). Functional analysis will probably expand with the recent application of the CRISPR/Cas9 system, which will provide the tools required to study the function of lncRNAs in genome-wide studies. These unsupervised studies will be crucial to functionally annotate the role of lncRNAs in cancer and shed further light on the regulation of the underlying molecular events (Han et al., 2014; Ho et al., 2015).

## lncRNA and miRNA Interplay

**FIGURE 1:**
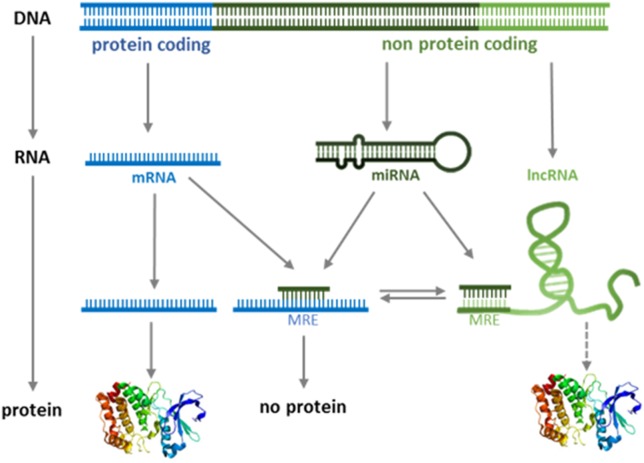
Modified version of the central dogma of molecular biology. The classical “DNA-RNA-protein” pathway is extended by functional role of ncRNAs.

Different lncRNAs are known to interact with DNA, RNA, and proteins; therefore, the functions of the class of lncRNAs appear to be pleiotropic, ranging from chromatin remodeling to the regulation of transcription, splicing, and translation (Wang and Chang, 2011; Fok et al., 2017; Kopp and Mendell, 2018). A subclass of lncRNA has recently been shown to regulate gene expression in *trans* by acting as miRNA “sponges” (Karreth et al., 2011; Salmena et al., 2011; Tay et al., 2011; Karreth and Pandolfi, 2013; Su et al., 2013; Yoon et al., 2014; Ulitsky, 2018). These lncRNAs belong to a group of RNAs named ceRNA (competitor of endogenous RNA) (Tay et al., 2014; Thomson and Dinger, 2016; Zhong et al., 2018). While there is no unifying definition of ceRNAs, their function as miRNA “sponges” minimally requires their cytoplasmic localization and the presence of MRE in their sequence. ceRNAs contain MREs for one or multiple miRNAs, and binding is thought to sequester miRNAs and thereby enable translation of endogenous miRNA targets (Figure 1).

**FIGURE 2:**
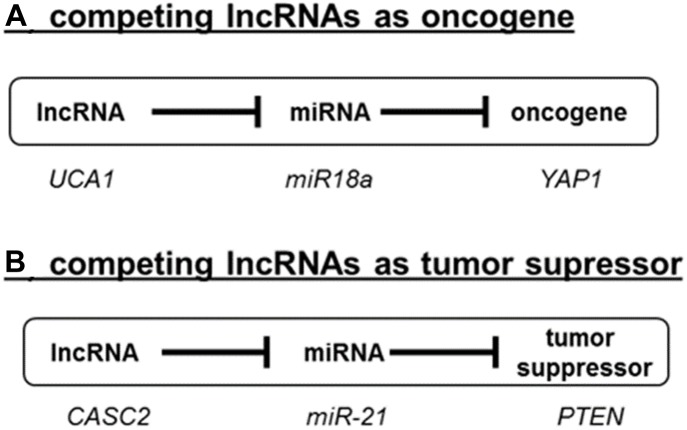
Competing lncRNAs (ceRNAs) in cancer. LncRNAs can act either as oncogenes **(A)** or as tumor suppressor **(B)** as exemplified by the role UCA1 and CASC2. A detailed literature survey of the reported roles ceRNAs in cancer is summarized in Supplementary Table S1.

In recent years, this concept has been described for the expression of different genes involved in tumor progression (de Giorgio et al., 2013; Cheng et al., 2015; Wang et al., 2016; Song et al., 2017). An example of an oncogenic lncRNA is UCA1, which controls the availability of miR-18a and thereby determines the expression of the oncogene YAP1 (Zhu et al., 2018; Figure 2A). In contrast, the tumor suppressor gene PTEN is regulated by the lncRNA CCAT2 by acting as a competing RNA for miR-21 (Figure 2B; Xie et al., 2017). In recent years, many more ceRNAs have been described in cancer. We have summarized the known lncRNA/miRNA/mRNA combinations in Supplementary Table S1.

In addition to sequestering miRNAs, lncRNAs have also been reported to compete with miRNAs by binding directly to mRNAs (Faghihi et al., 2010). miRNAs have been reported to induce destabilization of lncRNAs, yet some lncRNAs contain miRNAs precursors (Cui et al., 2016). This suggests a complex interplay between lncRNAs and miRNAs, which ultimately determines stability and translation of protein-coding mRNAs. Notably, recent studies have revealed that ceRNAs have significant roles in cancer pathogenesis (Cheng et al., 2015; Wang et al., 2016). For example, alterations in the expression of key factors in oncogenic signaling pathways, like BRAF, have been linked to changes in the level of ceRNAs (Ergun and Oztuzcu, 2015; Karreth et al., 2015). We are just starting to understand these complex molecular interactions, their place in functional regulatory networks controlling cellular processes, and their implications in cancer (Liz and Esteller, 2016; Yang et al., 2016; Cao et al., 2017; Chan and Tay, 2018).

## Proposal: Tissue-Specific Interplay Between miRNA and lncRNA Supports Context-Dependent Cell Signaling

Currently, the mechanisms involved in cancer origin and progression have been elaborated according to the alterations found in protein-coding genes, barely 2% of the translated genome. Following this approach, most of the described alterations merge into few biochemical routes such as the PI3K/AKT/mTOR or RAS/MAPK pathways. However, the outcome of these alterations is sometimes tissue-specific and cell-context-dependent. It is, for example, still unclear how certain oncogenic mutations progress to tumor formation only in a particular set of tissues. There are clear examples with germline mutations in genes like adenomatous polyposis coli (APC), cadherin 1 (CDH1), BRCA1, von Hippel-Lindau tumor suppressor (VHL), and ataxia telangiectasia mutated (ATM) which are causative for the development of cancer in specific types of tissue (Schneider et al., 2017). Another paradigmatic example is the initial good response to specific inhibitors in melanomas harboring the BRAF^V600E^ mutation, compared to the protumor effect of the same inhibitors in colon adenocarcinomas carrying similar BRAF mutations (Prahallad et al., 2012; Sclafani et al., 2013). Finally, the development of metastases in different types of cancer is often restricted to certain organs (organotropic metastasis), and even clonal subpopulations within the primary tumor display preferences for certain organs (Obenauf and Massague, 2015).

**FIGURE 3:**
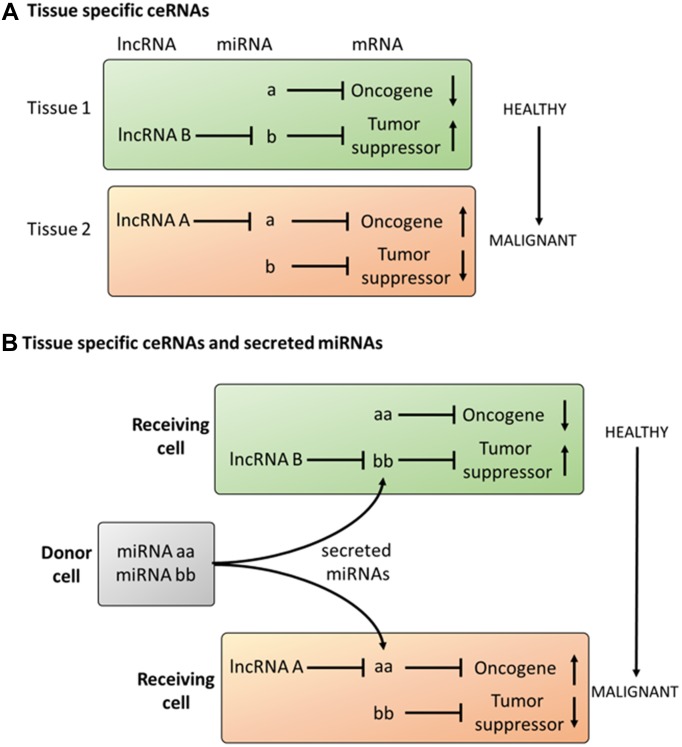
Proposal on the role of tissue specific ceRNAs in cancer **(A)** Tissue specific expression of lncRNAs can induce malignant transformation in cells expressing an equal set of miRNAs and mRNAs. **(B)** Tissue specific response to secreted miRNAs dependent on the expression profile of lncRNAs.

Therefore, in addition to the classical oncogenic signaling pathways (PI3K/AKT/mTOR or RAS/MAPK), additional layers of regulation must exist, to explain tissue- and organ-specific processes. We hypothesize that these cell-type-specific outcomes may be caused by interplay between lncRNAs and miRNAs. Building on the concept of ceRNAs, the tissue and cell-type-specific expression of lncRNAs might be a key mechanism to support tissue-specific regulation of oncogenic signaling pathways. In this sense, the translational profile (i.e., proteome) of miRNA-regulated mRNAs can be controlled by the cell-type-specific expression of lncRNAs. Consequently, the interplay between lncRNAs and miRNAs might affect signaling cascades by regulating the abundance of proteins within these pathways in a cell-type-specific manner (Figure 3A).

This core mechanism, acting on the intracellular level, can also be extended to communication between different cells. This is because miRNAs not only act in the cells in which they are transcribed but can also be transferred into different cells (intercellular level). In this regard, the interplay between miRNAs and lncRNAs suggests many parallelisms to ligand–receptor interactions. Ligands like cytokines, hormones and growth factors are released as soluble factors by the donor cell, selectively interact with the target cell receptors and activate signaling cascades which ultimately alter the phenotype of the target cell. miRNAs have been shown to be released into the extracellular space, and lncRNAs are expressed in an organ-, tissue- or cell-type-specific manner. As described above, the abundance of lncRNAs may ultimately determine the effect of miRNAs on the expression of protein-coding genes. Therefore, as occurs in the ligand-receptor model, exposure to the same miRNA may result in cell-type-specific alterations dependent on the expression of lncRNAs (Figure 3B).

In summary, the interplay between lncRNAs and miRNAs at an intra- and intercellular level may provide a framework for understanding context-specific phenomena in cancer. In the following section, we will exemplify these putative mechanisms in two concrete cases.

## Intracellular Interplay Between lncRNAs and miRNAs – BRAF^V600E^ in Colon Adenocarcinoma Vs Melanoma

**FIGURE 4:**
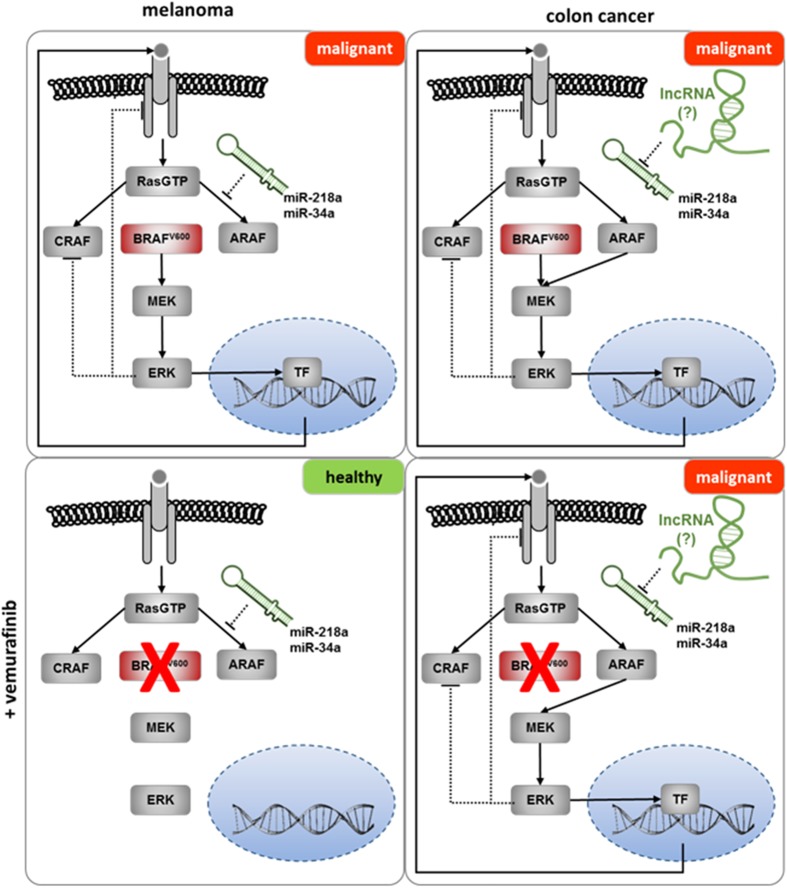
The potential role of lncRNAs in the failure of Vemurafenib in the treatment of colon cancer. Upper panel: BRAF^V600E^ mutation induces malignant transformation in melanoma and colon cancer. Lower panel (left): treatment with Vemurafenib inhibits malignant transformation in melanoma. Bypassing the inhibition of BRAF^V600E^ by Vemurafenib might be prevented by miRNAs (e.g., miR-34a or miR-218a) inhibiting the expression of ARAF (Lal et al., 2011; Agarwal et al., 2015). Lower panel (right): sequestering of the ARAF controlling miRNA(s) by lncRNAs in colon cancer enables the bypass of Vemurafenib inhibition, resulting in malignant transformation.

Transduction of a signal from an activated receptor is dependent on the levels of kinases and phosphatases in these pathways and is regulated by positive and negative feedback loops. Alterations in the stoichiometry of factors involved in signaling cascades can be crucial for the ultimate cellular effect. A paradigmatic example for such alterations in the same oncogenic pathway is described for the BRAF^V600E^ mutation. While treatment of BRAF^V600E^–bearing tumors with BRAF inhibitors has a good response in melanomas, a protumor effect has been described in colon adenocarcinomas. It has been proposed that the local feedback in the different cell-signaling pathways could partly explain this unexpected contradictory effect (Schneider et al., 2017). In this respect, miRNAs have been described as key players in fine-tuning the expression of proteins involved in the Ras/Raf signaling pathway (reviewed in Masliah-Planchon et al., 2016). However, the expression of the majority of these miRNAs is not specific for single tissues; therefore, additional layers of regulation must exist, to explain the different responses. We propose that the tissue-specific interplay between ncRNAs might partly explain the different sensitivity to BRAF inhibitors in colon adenocarcinoma and melanoma harboring the same oncogenic mutation in BRAF (Figure 4).

## Intercellular Interplay Between lncRNAs and miRNAs – Organotropic Metastasis

miRNAs are released into the extracellular space and transferred to target cells. lncRNAs (the “receptors”) show organ-, tissue- and cell-type-specific expression. Binding of miRNAs to lncRNAs (akin to a ligand-receptor complex) occurs via a sequence-specific MRE within the lncRNA. Finally, this interaction results in miRNA sequestration and/or degradation of miRNA or lncRNA. As the ligand-receptor model would predict, these interactions should result in the activation of signaling cascades enabling significant alterations in biochemical and cellular functions.

Applying this model to cancer, the mechanisms underlying organotropic metastasis might be explained in part by the interplay between miRNAs and lncRNAs. Even though there is no direct experimental evidence for this hypothesis, there are theoretical possibilities for how this interplay could contribute to the development of site-specific metastasis.

**FIGURE 5:**
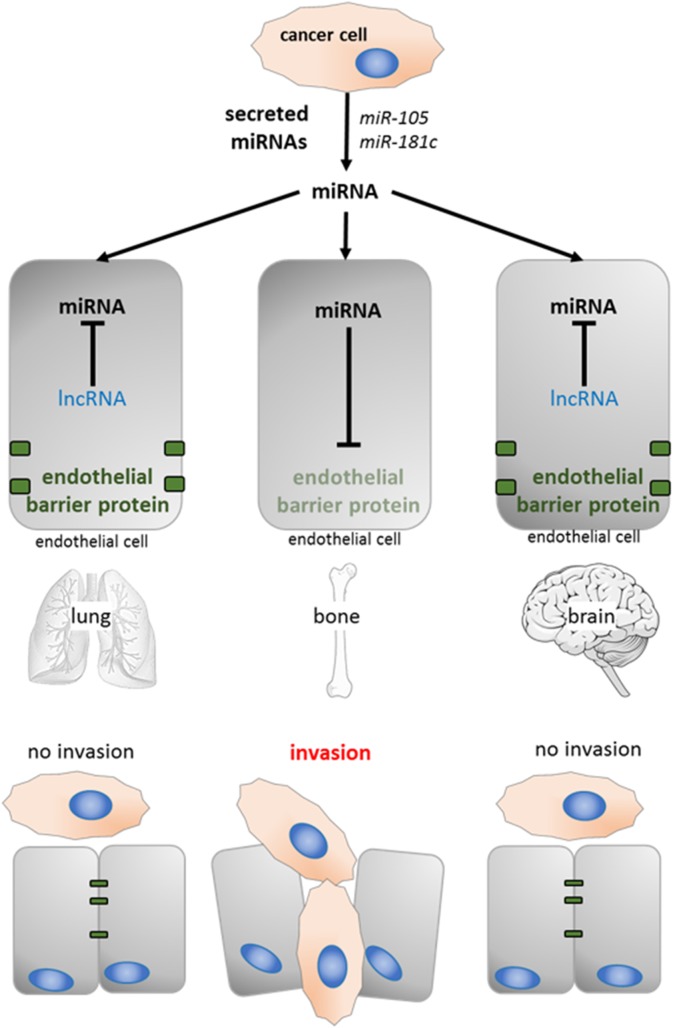
The potential role of lncRNAs in organotropic metastasis. Cancer cell secreted miRNA (e.g., miR-105 and miR181c prevent the expression of proteins with barrier function in endothelial cells Zhou et al., 2014; Tominaga et al., 2015). The presence of tissue specific competing lncRNAs can sequester those miRNAs (lung and brain), the endothelial barrier is maintained and cancer cells are not able to invade.

High levels of secreted miRNAs from cancer cells have been reported for almost all types of tumor cells. Upon arrival of a disseminated tumor cell to a distant tissue (e.g., lung, bone, or brain), these miRNAs are first taken up by tissue-specific endothelial cells. Transfer of miRNAs to endothelial cells in turn has been shown to alter the expression of proteins required for maintenance of the endothelial barrier (Zhou et al., 2014; Tominaga et al., 2015). Therefore, the expression profile of competing lncRNAs within endothelial cells might determine if the barrier function can be sustained in the presence of exogenous miRNAs, and metastasis will be favored in organs in which ceRNAs are absent (Figure 5).

In addition, aberrant expression of lncRNAs has been reported in different tumor types, and differential cell-to-cell expression has even been observed in certain tumor types (Lv et al., 2016). Therefore, the presence of sponging lncRNAs might alter (a) the miRNA secretome of tumor cells, and (b) the responsiveness to secreted miRNAs from other cells. The latter case in particular might account for clonal populations of tumor cells with site-specific patterns of metastasis (Obenauf and Massague, 2015).

It is tempting to speculate that the interplay between lncRNAs and miRNAs combined with the tissue- or clone-specific expression of lncRNAs might favor the formation of metastatic niches in an organ- or tissue-specific manner. However, future detailed studies will be required to prove this hypothesis.

## Future Directions

In our opinion, the continuous accumulation of large amounts of data by RNAseq of whole cancer transcriptomes or extracellular miRNAs might be only partially helpful to move forward. We propose exploring the mechanistic basics in cell-culture-based systems or even model organisms with a reduced complexity. Models derived from these studies could later be used to predict how the extracellular miRNA composition combined with a distinct set of intracellular lncRNAs might impact on disease progression.

In summary, current limitations in our understanding on the molecular mechanisms of cancer might be due to the fact that, until now, only 2% of the genome has been taken into account. Therefore, future studies should aim at expanding our current view of cancer by including the role of ncRNAs in the interpretation of cancer as a multifactorial disease. The proposed model, combining lncRNA-miRNA interactions with intercellular communication might be particularly helpful in understanding the tissue-specificity of many cancers, hitherto one of the least understood phenomena of cancer.

## Data Availability

Publicly available datasets were analyzed in this study. This data can be found here: https://www.ncbi.nlm.nih.gov/pubmed/.

## Author Contributions

All authors listed have made a substantial, direct and intellectual contribution to the work, and approved it for publication.

## Conflict of Interest Statement

The authors declare that the research was conducted in the absence of any commercial or financial relationships that could be construed as a potential conflict of interest.
