# Supplementary material for: Interplay Between ncRNAs and Cellular Communication: A Proposal for Understanding Cell-Specific Signaling Pathways
Source: Front Genet. 2019 Apr 2;10:281. doi: 10.3389/fgene.2019.00281 (PMC6454836; doi:10.3389/fgene.2019.00281)
Supplement: Supplementary file 1 [file Table_1.pdf]

Table S1: Participation of ceRNAs in cancer.

lncRNA	miRNA	mRNA	type of cancer	PMID
AFAP1-AS1	miR-146b-5p	EGFR	pancreatic cancer	(Zhou et al., 2018a)
ATB	miR-590-5p	YAP1	melanoma	(Mou et al., 2018)
ATB	miR-141-3p	TGFβ	Gastric cancer	(Lei et al., 2017b)
ATB	miR-200a	TGFB2	Glioma	(Ma et al., 2016)
BARD1 9'L	miR-203<br>miR-101	BARD1	Breast, Lung, Prostate (cancer cell lines)	(Pilyugin and Irminger-Finger, 2014)
BC032469	miR-1207-5p	hTERT	Gastric Cancer	(Lu et al., 2016b)
CASC2	miR-18a	PIAS3	Colorectal cancer	(Huang et al., 2016)
CASC2	miR-21	PTEN	cervical cancer	(Feng et al., 2017)
CASC2	miR-367	FBXW7	hepatocellular carcinoma	(Wang et al., 2017b)
CCAT1	miR-155	C-MYC	Acute Myeloid Laukemia	(Chen et al., 2016a)
CCAT1	miR-181b	FGFR3<br>PDGFRα	Glioma	(Cui et al., 2017)
CCAT1	miR-410	ITPKB	Colon cancer	(Li et al., 2017a)
CCAT1	miR-143	FTC-133	thyroid carcinoma	(Yang et al., 2018c)
CCAT1	miR-33a	n.d.	Melanoma	(Lv et al., 2018)
CCAT2	miR-424	n.d.	Ovarian Cancer	(Hua et al., 2018)
CCR492	Let-7	C-MYC	MEFs	(Maldotti et al., 2016)
CRNDE	miR-451	n.d.	Multiple Myeloma	(Meng et al., 2017)
CTNNAP1	miR-141	CTNNA1	Colorectal Cancer	(Chen et al., 2016b)
DANCR	miR-335-5p,<br>miR-1972	ROCK1	osteosarcoma	(Wang et al., 2018d)
DANCR	miR-149	MSI2	bladder cancer	(Zhan et al., 2018)
FAL1	miR-1236	n.d.	hepatocellular carcinoma	(Li et al., 2018a)
FER1L4	miR-106a-5p	n.d.	Colon Cancer	(Yue et al., 2015)
FER1L4	miR-106a-5p	PTEN<br>RB1<br>RUNX1<br>VEGFA<br>CDKN1A<br>E2F1,<br>HIPK3<br>IL-10,<br>PAK7	Gastric Cancer	(Xia et al., 2014)
FLVCR1-AS1	miR-573	E2F 3	lung cancer	(Gao et al., 2018b)
FOXD2-AS1	miR-143	ABCC3	bladder cancer	(An et al., 2018)
FOXD2-AS1	miR-485-5p	KLK7	papillary thyroid cancer	(Zhang et al., 2018e)
FOXD2-AS1	miR-185-5p	CCND2	glioma malignancy	(Shen et al., 2018a)
FOXD2-AS1	miR-363-5p	S100A1	nasopharyngeal carcinoma	(Chen et al., 2018a)
FOXD2-AS1	miR-206	ANXA2	hepatocellular carcinoma	(Chang et al., 2018b)
FOXD2-AS1	miR-185-5p	cdc42	Colorectal cancer	(Zhu et al., 2018b)

FTH1P3	miR-224-5p	Fizzled 5	Oral squamous cell carcinoma	(Zhang, 2017b)
GACAT3	miR-497	CCND2	breast cancer	(Zhong et al., 2018)
GAS5	MiR-23a	ATG3	Breast Cancer	(Gu et al., 2018)
GAS5	miR-135b	n.d.	Non-Small Cell Lung Cancer	(Xue et al., 2017)
GAS5	196a-5p	FOXO1	triple negative breast cancer	(Li et al., 2018c)
H1	miR-138	EZH2	oral squamous cell carcinoma	(Hong et al., 2018)
H19	Let-7a/b	IL-6	Cholangiocarcinoma	(Wang et al., 2016d)
H19	miR-342-3p	FOXM1	Gallbladder cancer	(Wang et al., 2016a)
H19	miR-17-5p	YES1	Thyroid cancer	(Liu et al., 2016a)
H19	miR-200 family	ZEB1, ZEB2	Osteosarcoma	(Li et al., 2016a)
H19	miR-138 miR-200a	VIM, ZEB1, ZEB2	Colorectal Cancer	(Liang et al., 2015)
H19	miR-29b-3p	DNMT3B	bladder cancer	(Lv et al., 2017)
HCAL	miR-15a miR-196a miR-196b	LAPTM4B	hepatocellular Carcinoma	(Xie et al., 2017)
HIF1A-AS2	miR-129-5p	DNMT3A	colorectal cancer	(Lin et al., 2018)
HNF1A-AS1	miR-30b-5p	ATG5	Hepatocellular Carcinoma	(Liu et al., 2016b)
HNF1A-AS1	miR-34a	SIRT1,p53	colon cancer	(Fang et al., 2017a)
HOTAIR	miR-138, miR-200c miR-204 miR-217	ADAM9 CCND2 EZH2 VEGFA VIM ZEB1 ZEB2	Renal cell carcinoma	(Ding et al., 2018b)
HOTAIR	miR-23b-3p	ZEB1	hepatocellular carcinoma	(Yang et al., 2018b)
HOTAIR	miR-217	HIF1μα	Renal cell carcinoma	(Hong et al., 2017)
HOTAIR	miR-373	RAB22A	Ovarian Cancer	(Zhang et al., 2016)
HOTAIR	miR-141	SKA2	Glioma	(Bian et al., 2016)
HOTAIR	miR-331-3p	HER2	Gastric Cancer	(Liu et al., 2014)
HOTAIR	miR-125 miR-143	HK2	oesophageal squamous cell carcinoma	(Ma et al., 2017)
HOTAIR	miR-206	CCND1 CCND2	Ovarian Cancer	(Chang et al., 2018a)
HOXA11-AS	miR-454-3p	Stat3	lung adenocarcinoma	(Zhao et al., 2018)
HOXA11-AS	miR-214-3p	EZH2	glioma	(Xu et al., 2017a)
HOXA-AS2	miR-520c-3p	S100A4	Papillary Thyroid Cancer	(Xia et al., 2018)
HOXD-AS1	miR-130a	E2F8	glioma	(Chen et al., 2018b)
HOXD-AS1	miR-130a-3p	SOX4	hepatocellular carcinoma	(Wang et al., 2017a)
HULC	miR-372	CXCR4	Cholangiocarcinoma	(Wang et al., 2016d)

	miR-373			
HULC	miR-200a-3p	ZEB1	Hepatocellular carcinoma	(Li et al., 2016b)
KRTAP5-AS1 TUBB2A	miR-596 miR-3620-3p	CLDN4	gastric cancer	(Song et al., 2017)
LIFR-AS1	miR-29a	TNFAIP3	colorectal cancer	(Liu et al., 2018c)
LINC00152	miRNA-206	NRP1	colorectal cancer	(Chen et al., 2018c)
LINC00174	miR-1910-3p	TAZ	colorectal carcinoma	(Shen et al., 2018b)
LINC00511	miR-765	LAMC2	tongue squamous cell carcinoma	(Ding et al., 2018a)
LINC00668	miR-297	n.d.	oral squamous cell carcinoma	(Zhang, 2017a)
LINC00858	miR-422	KLK4	Non-small Cell Lung Cancer	(Zhu et al., 2017)
Linc00974	miR-642	KRT19	Hepatocellular Carcinoma	(Tang et al., 2014)
linc01561	miR-145-5p	MMP11	breast cancer	(Jiang et al., 2018b)
LOC728196	miR-513c	TCF7	glioma carcinogenesis	(Wang et al., 2018c)
MALAT1	miR-211	PHF19	ovarian carcinoma	(Tao et al., 2018)
MALAT1	miR-143-3p	ZEB1	Hepatocellular Carcinoma	(Chen et al., 2017b)
MALAT1	miR-183	ITG1	melanoma	(Sun et al., 2017b)
MALAT1	miR-202	GLI2	Gastric Cancer	(Zhang et al., 2017d)
MALAT1	miR-363-3p	MCL-1	Gallbladder Cancer	(Wang et al., 2016b)
MALAT1	miR-22	MMP14 SNAIL1	Melanoma	(Luan et al., 2016)
MALAT1	miR-206	ANXA2 KRAS	Gallbladder cancer	(Wang et al., 2016c)
MALAT1	miR-1	CDC42	Breast Cancer	(Chou et al., 2016)
MALAT1	miR-145	Nd.	Cervical Cancer	(Lu et al., 2016a)
MALAT1	miR-320a	FOXM1		(Sun et al., 2017a)
MALAT1	miR-211	PHF19	ovarian carcinoma.	(Tao et al., 2018)
MALAT1	miR-195	EGFR	hepatoma cells	(Liu et al., 2018a)
MALAT1	miR-125b	STAT3	oral squamous cell carcinoma	(Chang and Hu, 2018)
MEG3	miR-181	BCL2	Gastric Cancer	(Peng et al., 2015)
MEG3	miR-19a	PTEN	glioma	(Qin et al., 2017)
MEG3	hsa-miR-9	E-cadherin FOXO1	Esophageal Cancer	(Dong et al., 2017)
MIAT	MiR-29c	LOXL2	Clear Cell Renal Cell Carcinoma	(Qu et al., 2018)
MKI67IP-3	Let-7e	IKBβ	Inflamatory response	(Lin et al., 2017)
MNX1-AS1	miR-218-5p	SEC61A1	colon adenocarcinoma.	(Ye et al., 2018)
MT1JP	miR-214-3p	RUNX3	Gastric Cancer	(Xu et al., 2018)
NEAT1	miR-186-5p	HIF-1α	osteosarcoma	(Tan and Zhao, 2018)
NEAT1	miR-339-5p	TGF-β1	osteosarcoma	(Zhang et al., 2019)
NEAT1	mir-139-5p	TGF-β1	hepatocellular carcinoma	(Tu et al., 2018)
NEAT1	miR-485	STAT3	hepatocellular carcinoma	(Zhang et al., 2018d)

NEAT1	miR-506	STAT3	gastric cancer	(Tan et al., 2018)
NEAT1	miR-448	ZEB1	breast cancer	(Jiang et al., 2018c)
NEAT1	mir-98-5p	MAPK6	non-small-cell lung cancer	(Wu et al., 2017a)
NEAT1	miR-9-5p	n.d.	cervical cancer	(Xie et al., 2018b)
NEAT1	miR-129	CTBP2	Esophageal Squamous Cell Carcinoma	(Li et al., 2017h)
NEAT1	miR-377-3p	E2F3	Non-small cell lung cancer	(Sun et al., 2016)
NEAT1	miR-382-3p	ROCK1	ovarian cancer	(Liu et al., 2018g)
NEAT1	Mir-193a-3p	USF1	Lung Adenocarcinoma	(Xiong et al., 2018)
NEAT1	miR-382-3p	ROCK1	Ovarian cancer	(Liu et al., 2018g)
NeD125	miR-19a-3p, miR-19b-3p miR-106a-5p	CDK6 MYCN SNCAIP KDM6A	Medulloblastoma	(Laneve et al., 2017)
NNT-AS1	miR-129-5p	n.d.	lung cancer	(Shen and Jiang, 2018)
NNT-AS1	miR-142-3p	ZEB1	breast cancer	(Zhang et al., 2018e)
NNT-AS1	miR-142-3p	ZEB1	breast cancer	(Li et al., 2018d)
NORAD	miR-590-3p	SIP1	cervical cancer	(Huo et al., 2018)
NORAD	miR-125a-3p	RhoA	pancreatic cancer	(Li et al., 2017c)
OIP5-AS1	miR-186a-5p	ZEB1	hepatoblastoma	(Zhang et al., 2018g)
OIP5-AS1.	miRNA-129-5p	SOX2	breast cancer	(Zeng et al., 2018)
PAGBC	miR-133b miR-511	n.d.	gallbladder tumorigenesis	(Wu et al., 2017b)
PCAT-1	miR-129-5p	HMGB1	hepatocellular carcinoma	(Zhang et al., 2017a)
PCAT7	miR-134-5p	ELF2	nasopharyngeal carcinoma	(Liu et al., 2017c)
PTENP1	miR-106b miR-93	PTEN	Gastric cancer	(Zhang et al., 2017b)
PTENP1	miR-19b	PTEN	breast cancer	(Li et al., 2017f)
PVT1	microRNA-30a	IGFR1.	papillary thyroid carcinoma	(Feng et al., 2018)
PVT1	miR-199a-5p	HIF1α	non-small cell lung cancer	(Wang et al., 2018a)
PVT1	miR-424	n.d.	Cervical Cancer	(Gao et al., 2017)
PVT1-5	miR-126	SLC7A5	lung cancer	(Li et al., 2018b)
ROR	miR-145	N.d.	Colon Cancer	(Zhou et al., 2016)
ROR	miR-145	Nanog	Pancreatic Cancer	(Gao et al., 2016)
RoR	miR-145	MUC1	triple-negative breast cancer	(Ma et al., 2018)
ROR	miR-145	Oct4 Sox2 Nanog	Colon cancer	(Yan and Sun, 2018)
ROR	miR-145	FSCN1	esophageal squamous cell carcinoma	(Shang et al., 2018)
ROR	miR-145	Oct4	prostate cancer	(Liu et al., 2017b)
ROR	miR-145	n.d.	Endometrial Cancer	(Zhou et al., 2014)

RP11-79H23.3	miR-107	PTEN	Bladder Cancer.	(Chi et al., 2018)
RP4	miR-7-5p	SH3GLB1	colorectal cancer	(Liu et al., 2018d)
RSU1P2	let-7a	n.d.	cervical cancer	(Liu et al., 2017a)
SNHG1	miR-338	CST3	Esophageal cancer	(Yan et al., 2017)
SNHG1	miR-199a-3p	CDK7	Prostate cancer	(Li et al., 2017e)
SNHG1	miR-577	n.d.	osteosarcoma	(Jiang et al., 2018d)
SNHG1	miR-326	NOB1	osteosarcoma	(Wang et al., 2018b)
SNHG12	miR-199a/b-5p	MLK3	Hepatocellular Carcinoma	(Lan et al., 2017)
SNHG12	miR-195-5p	Notch2	osteosarcoma	(Zhou et al., 2018b)
SNHG16	miR-98	E2F5	Breast cancer	(Cai et al., 2017)
SNHG16	miR-20a-5p	E2F1	glioma	(Yang et al., 2018a)
SNHG5	miR-32	KLF4	Gastric cancer	(Zhao et al., 2017)
SNHG6-003	miR-26a/b	TAK1	Hepatocellular carcinoma	(Cao et al., 2017)
SNHG7	miR-193b	FAIM2-	non-small cell lung cancer	(She et al., 2018)
SPRY4-IT1	miR-101-3p	n.d.	colorectal cancer	(Jin et al., 2017)
STARD1	miR-9<br>miR-10b<br>miR-125	CDH5<br>HOXD1,<br>HOXD10	Breast Cancer	(Li et al., 2016c)
TINCR	miR-544a	FBXW7	lung cancer	(Liu et al., 2018f)
TP73-AS1	miR-449a	EZH2	non-small cell lung cancer	(Zhang et al., 2018a)
TP73-AS1	miR-124	iASPP	Glioma	(Xiao et al., 2018)
TP73-AS1	miR-200a	TFAM	breast cancer	(Yao et al., 2018)
TP73-AS1	miR-200a	ZEB1	breast cancer	(Zou et al., 2018)
TP73-AS1	miR-200a	HMGB1	hepatocellular carcinoma	(Li et al., 2017g)
TP73-AS1	miR-449a	EZH2	non-small cell lung cancer	(Zhang et al., 2018a)
TP73-AS1	miR-142	HMGB1	Brain Glioma	(Zhang et al., 2018c)
TUG1	mir-142-3p	ZEB1	Hepatocellular Carcinoma	(He et al., 2018)
TUG1	miR-335-5p	n.d.	Osteosarcoma	(Zhang et al., 2017b)
TUG1	miR-9-5p	POU2F1	Osteosarcoma	(Xie et al., 2016)
TUG1	miR-186	CPEB2	colorectal cancer	(Li et al., 2017b)
TUG1	miR-145	n.d.	papillary thyroid cancer	(Lei et al., 2017a)
TUG1	miR-212-3p	FOXA1	osteosarcoma	(Xie et al., 2018a)
TUSC7	miR-211	CDK6	Colorectal Cancer	(Xu et al., 2017b)
UCA1	miR-195	ARL2	Bladder Cancer	(Li et al., 2017d)
UCA1	miR-16	MDR1	Chronic Myeloid Leukemia	(Xiao et al., 2017)
UCA1	miR-204	SOX4	Esophageal Cancer	(Jiao et al., 2016)
UCA1	miR-193a	HMGB1	lung cancer cell	(Wu and Zhou, 2018)
UCA1	miR-193a-3p	ERBB4	non-small cell lung cancer	(Nie et al., 2016)
UCA1	miR-184	SF1	oral squamous cell carcinoma	(Fang et al., 2017b)
UCA1	miR-196a-5p	CREB	bladder cancer	(Pan et al., 2016)

UCA1	hsa-miR-145	ZEB1<br>ZEB2<br>FSCN1	bladder cancer	(Xue et al., 2016)
UCA1	miR-204-5p	ZEB1<br>COL5 A1	glioma	(Liang et al., 2018)
UCA1	miR-204	Sox4	esophageal cancer	(Jiao et al., 2016)
UCA1	miR-16	MDR1	Myeloid Leukemia Cells	(Xiao et al., 2017)
UCA1	MiR-195	ARL2	Bladder Cancer	(Li et al., 2017d)
UCA1	miR-204	ATF2	prostate cancer	(Zhang et al., 2017c)
UCA1	miR129	SOX4	renal cell carcinoma	(Liu et al., 2018e)
UCA1	miR-125a	HK2	pediatric AML	(Zhang et al., 2018f)
UICLM	microRNA-215	ZEB2	colorectal cancer	(Chen et al., 2017a)
Unigene56159	miR-140-5p	SLUG	Hepatocellular carcinoma	(Lv et al., 2016)
USP16	miR-21<br>miR-590-5p	PTEN	Hepatocellular carcinoma	(Sui et al., 2017)
XIST	miR-34a	MET	thyroid cancer	(Liu et al., 2018b)
XIST	miR-200a	Fus	cervical cancer	(Zhu et al., 2018a)
XIST	miR-137	PXN	non-small cell lung cancer	(Jiang et al., 2018a)
XIST	miR-185	TGF-β1	gastric cancer	(Zhang et al., 2018b)
XIST	miR-21-5p	PDCD4	osteosarcoma	(Zhang and Xia, 2017)
XLOC_006390	miR-338-3p<br>miR-331-3p	PKM2<br>EYA2<br>NRP2	cervical cancer	(Luan and Wang, 2018)
XLOC_008466	miR-874	MMP2<br>XIAP	Non-small Cell Lung Cancer	(Yang et al., 2017)
ZEB2-AS1	miR-204	HMGB1	pancreatic cancer	(Gao et al., 2018a)

References (Table S1.)

An, Q., Zhou, L., and Xu, N. (2018). Long noncoding RNA FOXD2-AS1 accelerates the gemcitabine-resistance of bladder cancer by sponging miR-143. *Biomed Pharmacother* 103**,** 415-420. doi: 10.1016/j.biopha.2018.03.138.

Bian, E.B., Ma, C.C., He, X.J., Wang, C., Zong, G., Wang, H.L., et al. (2016). Epigenetic modification of miR-141 regulates SKA2 by an endogenous 'sponge' HOTAIR in glioma. *Oncotarget* 7(21)**,** 30610-30625. doi: 10.18632/oncotarget.8895.

Cai, C., Huo, Q., Wang, X., Chen, B., and Yang, Q. (2017). SNHG16 contributes to breast cancer cell migration by competitively binding miR-98 with E2F5. *Biochem Biophys Res Commun* 485(2)**,** 272-278. doi: 10.1016/j.bbrc.2017.02.094.

Cao, C., Zhang, T., Zhang, D., Xie, L., Zou, X., Lei, L., et al. (2017). The long non-coding RNA, SNHG6-003, functions as a competing endogenous RNA to promote the progression of hepatocellular carcinoma. *Oncogene* 36(8)**,** 1112-1122. doi: 10.1038/onc.2016.278.

Cui, B., Li, B., Liu, Q., and Cui, Y. (2017). lncRNA CCAT1 Promotes Glioma Tumorigenesis by Sponging miR-181b. *J Cell Biochem* 118(12)**,** 4548-4557. doi: 10.1002/jcb.26116.

Chang, L., Guo, R., Yuan, Z., Shi, H., and Zhang, D. (2018a). LncRNA HOTAIR Regulates CCND1 and CCND2 Expression by Sponging miR-206 in Ovarian Cancer. *Cell Physiol Biochem* 49(4)**,** 1289-1303. doi: 10.1159/000493408.

Chang, S.M., and Hu, W.W. (2018). Long non-coding RNA MALAT1 promotes oral squamous cell carcinoma development via microRNA-125b/STAT3 axis. *J Cell Physiol* 233(4)**,** 3384-3396. doi: 10.1002/jcp.26185.

Chang, Y., Zhang, J., Zhou, C., Qiu, G., Wang, G., Wang, S., et al. (2018b). Long non-coding RNA FOXD2-AS1 plays an oncogenic role in hepatocellular carcinoma by targeting miR206. *Oncol Rep* 40(6)**,** 3625-3634. doi: 10.3892/or.2018.6752.

Chen, D.L., Lu, Y.X., Zhang, J.X., Wei, X.L., Wang, F., Zeng, Z.L., et al. (2017a). Long non-coding RNA UICLM promotes colorectal cancer liver metastasis by acting as a ceRNA for microRNA-215 to regulate ZEB2 expression. *Theranostics* 7(19)**,** 4836-4849. doi: 10.7150/thno.20942.

Chen, G., Sun, W., Hua, X., Zeng, W., and Yang, L. (2018a). Long non-coding RNA FOXD2-AS1 aggravates nasopharyngeal carcinoma carcinogenesis by modulating miR-363-5p/S100A1 pathway. *Gene* 645**,** 76-84. doi: 10.1016/j.gene.2017.12.026.

Chen, L., Wang, W., Cao, L., Li, Z., and Wang, X. (2016a). Long Non-Coding RNA CCAT1 Acts as a Competing Endogenous RNA to Regulate Cell Growth and Differentiation in Acute Myeloid Leukemia. *Mol Cells* 39(4)**,** 330-336. doi: 10.14348/molcells.2016.2308.

Chen, L., Yao, H., Wang, K., and Liu, X. (2017b). Long Non-Coding RNA MALAT1 Regulates ZEB1 Expression by Sponging miR-143-3p and Promotes Hepatocellular Carcinoma Progression. *J Cell Biochem* 118(12)**,** 4836-4843. doi: 10.1002/jcb.26158.

Chen, X., Zhu, H., Wu, X., Xie, X., Huang, G., Xu, X., et al. (2016b). Downregulated pseudogene CTNNAP1 promote tumor growth in human cancer by downregulating its cognate gene CTNNA1 expression. *Oncotarget* 7(34)**,** 55518-55528. doi: 10.18632/oncotarget.10833.

Chen, Y., Zhao, F., Cui, D., Jiang, R., Chen, J., Huang, Q., et al. (2018b). HOXD-AS1/miR-130a sponge regulates glioma development by targeting E2F8. *Int J Cancer* 142(11)**,** 2313-2322. doi: 10.1002/ijc.31262.

Chen, Z.P., Wei, J.C., Wang, Q., Yang, P., Li, W.L., He, F., et al. (2018c). Long noncoding RNA 00152 functions as a competing endogenous RNA to regulate NRP1 expression by sponging with miRNA206 in colorectal cancer. *Int J Oncol* 53(3)**,** 1227-1236. doi: 10.3892/ijo.2018.4451.

Chi, H., Yang, R., Zheng, X., Zhang, L., Jiang, R., and Chen, J. (2018). LncRNA RP11-79H23.3 Functions as a Competing Endogenous RNA to Regulate PTEN Expression through Sponging hsa-miR-107 in the Development of Bladder Cancer. *Int J Mol Sci* 19(9). doi: 10.3390/ijms19092531.

Chou, J., Wang, B., Zheng, T., Li, X., Zheng, L., Hu, J., et al. (2016). MALAT1 induced migration and invasion of human breast cancer cells by competitively binding miR-1 with cdc42. *Biochem Biophys Res Commun* 472(1)**,** 262-269. doi: 10.1016/j.bbrc.2016.02.102.

Ding, J., Yang, C., and Yang, S. (2018a). LINC00511 interacts with miR-765 and modulates tongue squamous cell carcinoma progression by targeting LAMC2. *J Oral Pathol Med* 47(5)**,** 468-476. doi: 10.1111/jop.12677.

Ding, J., Yeh, C.R., Sun, Y., Lin, C., Chou, J., Ou, Z., et al. (2018b). Estrogen receptor beta promotes renal cell carcinoma progression via regulating LncRNA HOTAIR-miR-138/200c/204/217 associated CeRNA network. *Oncogene* 37(37)**,** 5037-5053. doi: 10.1038/s41388-018-0175-6.

Dong, Z., Zhang, A., Liu, S., Lu, F., Guo, Y., Zhang, G., et al. (2017). Aberrant Methylation-Mediated Silencing of lncRNA MEG3 Functions as a ceRNA in Esophageal Cancer. *Mol Cancer Res* 15(7)**,** 800-810. doi: 10.1158/1541-7786.MCR-16-0385.

Fang, C., Qiu, S., Sun, F., Li, W., Wang, Z., Yue, B., et al. (2017a). Long non-coding RNA HNF1A-AS1 mediated repression of miR-34a/SIRT1/p53 feedback loop promotes the metastatic progression of colon cancer by functioning as a competing endogenous RNA. *Cancer Lett* 410**,** 50-62. doi: 10.1016/j.canlet.2017.09.012.

Fang, Z., Zhao, J., Xie, W., Sun, Q., Wang, H., and Qiao, B. (2017b). LncRNA UCA1 promotes proliferation and cisplatin resistance of oral squamous cell carcinoma by sunppressing miR-184 expression. *Cancer Med* 6(12)**,** 2897-2908. doi: 10.1002/cam4.1253.

Feng, K., Liu, Y., Xu, L.J., Zhao, L.F., Jia, C.W., and Xu, M.Y. (2018). Long noncoding RNA PVT1 enhances the viability and invasion of papillary thyroid carcinoma cells by functioning as ceRNA of microRNA-30a through mediating expression of insulin like growth factor 1 receptor. *Biomed Pharmacother* 104**,** 686-698. doi: 10.1016/j.biopha.2018.05.078.

Feng, Y., Zou, W., Hu, C., Li, G., Zhou, S., He, Y., et al. (2017). Modulation of CASC2/miR-21/PTEN pathway sensitizes cervical cancer to cisplatin. *Arch Biochem Biophys* 623-624**,** 20-30. doi: 10.1016/j.abb.2017.05.001.

Gao, H., Gong, N., Ma, Z., Miao, X., Chen, J., Cao, Y., et al. (2018a). LncRNA ZEB2-AS1 promotes pancreatic cancer cell growth and invasion through regulating the miR-204/HMGB1 axis. *Int J Biol Macromol* 116**,** 545-551. doi: 10.1016/j.ijbiomac.2018.05.044.

Gao, S., Wang, P., Hua, Y., Xi, H., Meng, Z., Liu, T., et al. (2016). ROR functions as a ceRNA to regulate Nanog expression by sponging miR-145 and predicts poor prognosis in pancreatic cancer. *Oncotarget* 7(2)**,** 1608-1618. doi: 10.18632/oncotarget.6450.

Gao, X., Zhao, S., Yang, X., Zang, S., and Yuan, X. (2018b). Long non-coding RNA FLVCR1-AS1 contributes to the proliferation and invasion of lung cancer by sponging miR-573 to upregulate the expression of E2F transcription factor 3. *Biochem Biophys Res Commun* 505(3)**,** 931-938. doi: 10.1016/j.bbrc.2018.09.057.

Gao, Y.L., Zhao, Z.S., Zhang, M.Y., Han, L.J., Dong, Y.J., and Xu, B. (2017). Long Noncoding RNA PVT1 Facilitates Cervical Cancer Progression via Negative Regulating of miR-424. *Oncol Res* 25(8)**,** 1391-1398. doi: 10.3727/096504017X14881559833562.

Gu, J., Wang, Y., Wang, X., Zhou, D., Wang, X., Zhou, M., et al. (2018). Effect of the LncRNA GAS5-MiR-23a-ATG3 Axis in Regulating Autophagy in Patients with Breast Cancer. *Cell Physiol Biochem* 48(1)**,** 194-207. doi: 10.1159/000491718.

He, C., Liu, Z., Jin, L., Zhang, F., Peng, X., Xiao, Y., et al. (2018). lncRNA TUG1-Mediated Mir-142-3p Downregulation Contributes to Metastasis and the Epithelial-to-Mesenchymal Transition of Hepatocellular Carcinoma by Targeting ZEB1. *Cell Physiol Biochem* 48(5)**,** 1928-1941. doi: 10.1159/000492517.

Hong, Q., Li, O., Zheng, W., Xiao, W.Z., Zhang, L., Wu, D., et al. (2017). LncRNA HOTAIR regulates HIF-1alpha/AXL signaling through inhibition of miR-217 in renal cell carcinoma. *Cell Death Dis* 8(5)**,** e2772. doi: 10.1038/cddis.2017.181.

Hong, Y., He, H., Sui, W., Zhang, J., Zhang, S., and Yang, D. (2018). Long non-coding RNA H1 promotes cell proliferation and invasion by acting as a ceRNA of miR138 and releasing EZH2 in oral squamous cell carcinoma. *Int J Oncol* 52(3)**,** 901-912. doi: 10.3892/ijo.2018.4247.

Hua, F., Li, C.H., Chen, X.G., and Liu, X.P. (2018). Long Noncoding RNA CCAT2 Knockdown Suppresses Tumorous Progression by Sponging miR-424 in Epithelial Ovarian Cancer. *Oncol Res* 26(2)**,** 241-247. doi: 10.3727/096504017X14953948675412.

Huang, G., Wu, X., Li, S., Xu, X., Zhu, H., and Chen, X. (2016). The long noncoding RNA CASC2 functions as a competing endogenous RNA by sponging miR-18a in colorectal cancer. *Sci Rep* 6**,** 26524. doi: 10.1038/srep26524.

Huo, H., Tian, J., Wang, R., Li, Y., Qu, C., and Wang, N. (2018). Long non-coding RNA NORAD upregulate SIP1 expression to promote cell proliferation and invasion in cervical cancer. *Biomed Pharmacother* 106**,** 1454-1460. doi: 10.1016/j.biopha.2018.07.101.

Jiang, H., Zhang, H., Hu, X., and Li, W. (2018a). Knockdown of long non-coding RNA XIST inhibits cell viability and invasion by regulating miR-137/PXN axis in non-small cell lung cancer. *Int J Biol Macromol* 111**,** 623-631. doi: 10.1016/j.ijbiomac.2018.01.022.

Jiang, R., Zhao, C., Gao, B., Xu, J., Song, W., and Shi, P. (2018b). Mixomics analysis of breast cancer: Long non-coding RNA linc01561 acts as ceRNA involved in the progression of breast cancer. *Int J Biochem Cell Biol* 102**,** 1-9. doi: 10.1016/j.biocel.2018.06.003.

Jiang, X., Zhou, Y., Sun, A.J., and Xue, J.L. (2018c). NEAT1 contributes to breast cancer progression through modulating miR-448 and ZEB1. *J Cell Physiol* 233(11)**,** 8558-8566. doi: 10.1002/jcp.26470.

Jiang, Z., Jiang, C., and Fang, J. (2018d). Up-regulated lnc-SNHG1 contributes to osteosarcoma progression through sequestration of miR-577 and activation of WNT2B/Wnt/beta-catenin pathway. *Biochem Biophys Res Commun* 495(1)**,** 238-245. doi: 10.1016/j.bbrc.2017.11.012.

Jiao, C., Song, Z., Chen, J., Zhong, J., Cai, W., Tian, S., et al. (2016). lncRNA-UCA1 enhances cell proliferation through functioning as a ceRNA of Sox4 in esophageal cancer. *Oncol Rep* 36(5)**,** 2960-2966. doi: 10.3892/or.2016.5121.

Jin, J., Chu, Z., Ma, P., Meng, Y., and Yang, Y. (2017). Long non-coding RNA SPRY4-IT1 promotes proliferation and invasion by acting as a ceRNA of miR-101-3p in colorectal cancer cells. *Tumour Biol* 39(7)**,** 1010428317716250. doi: 10.1177/1010428317716250.

Lan, T., Ma, W., Hong, Z., Wu, L., Chen, X., and Yuan, Y. (2017). Long non-coding RNA small nucleolar RNA host gene 12 (SNHG12) promotes tumorigenesis and metastasis by targeting miR-199a/b-5p in hepatocellular carcinoma. *J Exp Clin Cancer Res* 36(1)**,** 11. doi: 10.1186/s13046-016-0486-9.

Laneve, P., Po, A., Favia, A., Legnini, I., Alfano, V., Rea, J., et al. (2017). The long noncoding RNA linc-NeD125 controls the expression of medulloblastoma driver genes by microRNA sponge activity. *Oncotarget* 8(19)**,** 31003-31015. doi: 10.18632/oncotarget.16049.

Lei, H., Gao, Y., and Xu, X. (2017a). LncRNA TUG1 influences papillary thyroid cancer cell proliferation, migration and EMT formation through targeting miR-145. *Acta Biochim Biophys Sin (Shanghai)* 49(7)**,** 588-597. doi: 10.1093/abbs/gmx047.

Lei, K., Liang, X., Gao, Y., Xu, B., Xu, Y., Li, Y., et al. (2017b). Lnc-ATB contributes to gastric cancer growth through a MiR-141-3p/TGFbeta2 feedback loop. *Biochem Biophys Res Commun* 484(3)**,** 514-521. doi: 10.1016/j.bbrc.2017.01.094.

Li, B., Mao, R., Liu, C., Zhang, W., Tang, Y., and Guo, Z. (2018a). LncRNA FAL1 promotes cell proliferation and migration by acting as a CeRNA of miR-1236 in hepatocellular carcinoma cells. *Life Sci* 197**,** 122-129. doi: 10.1016/j.lfs.2018.02.006.

Li, B., Shi, C., Zhao, J., and Li, B. (2017a). Long noncoding RNA CCAT1 functions as a ceRNA to antagonize the effect of miR-410 on the down-regulation of ITPKB in human HCT-116 and HCT-8 cells. *Oncotarget* 8(54)**,** 92855-92863. doi: 10.18632/oncotarget.21612.

Li, C., Gao, Y., Li, Y., and Ding, D. (2017b). TUG1 mediates methotrexate resistance in colorectal cancer via miR-186/CPEB2 axis. *Biochem Biophys Res Commun* 491(2)**,** 552-557. doi: 10.1016/j.bbrc.2017.03.042.

Li, H., Chen, S., Liu, J., Guo, X., Xiang, X., Dong, T., et al. (2018b). Long non-coding RNA PVT1-5 promotes cell proliferation by regulating miR-126/SLC7A5 axis in lung cancer. *Biochem Biophys Res Commun* 495(3)**,** 2350-2355. doi: 10.1016/j.bbrc.2017.12.114.

Li, H., Wang, X., Wen, C., Huo, Z., Wang, W., Zhan, Q., et al. (2017c). Long noncoding RNA NORAD, a novel competing endogenous RNA, enhances the hypoxia-induced epithelial-

mesenchymal transition to promote metastasis in pancreatic cancer. *Mol Cancer* 16(1), 169. doi: 10.1186/s12943-017-0738-0.
Li, H.J., Sun, X.M., Li, Z.K., Yin, Q.W., Pang, H., Pan, J.J., et al. (2017d). LncRNA UCA1 Promotes Mitochondrial Function of Bladder Cancer via the MiR-195/ARL2 Signaling Pathway. *Cell Physiol Biochem* 43(6), 2548-2561. doi: 10.1159/000484507.
Li, J., Zhang, Z., Xiong, L., Guo, C., Jiang, T., Zeng, L., et al. (2017e). SNHG1 lncRNA negatively regulates miR-199a-3p to enhance CDK7 expression and promote cell proliferation in prostate cancer. *Biochem Biophys Res Commun* 487(1), 146-152. doi: 10.1016/j.bbrc.2017.03.169.
Li, M., Chen, H., Zhao, Y., Gao, S., and Cheng, C. (2016a). H19 Functions as a ceRNA in Promoting Metastasis Through Decreasing miR-200s Activity in Osteosarcoma. *DNA Cell Biol* 35(5), 235-240. doi: 10.1089/dna.2015.3171.
Li, R.K., Gao, J., Guo, L.H., Huang, G.Q., and Luo, W.H. (2017f). PTENP1 acts as a ceRNA to regulate PTEN by sponging miR-19b and explores the biological role of PTENP1 in breast cancer. *Cancer Gene Ther* 24(7), 309-315. doi: 10.1038/cgt.2017.29.
Li, S., Huang, Y., Huang, Y., Fu, Y., Tang, D., Kang, R., et al. (2017g). The long non-coding RNA TP73-AS1 modulates HCC cell proliferation through miR-200a-dependent HMGB1/RAGE regulation. *J Exp Clin Cancer Res* 36(1), 51. doi: 10.1186/s13046-017-0519-z.
Li, S., Zhou, J., Wang, Z., Wang, P., Gao, X., and Wang, Y. (2018c). Long noncoding RNA GAS5 suppresses triple negative breast cancer progression through inhibition of proliferation and invasion by competitively binding miR-196a-5p. *Biomed Pharmacother* 104, 451-457. doi: 10.1016/j.biopha.2018.05.056.
Li, S.P., Xu, H.X., Yu, Y., He, J.D., Wang, Z., Xu, Y.J., et al. (2016b). LncRNA HULC enhances epithelial-mesenchymal transition to promote tumorigenesis and metastasis of hepatocellular carcinoma via the miR-200a-3p/ZEB1 signaling pathway. *Oncotarget* 7(27), 42431-42446. doi: 10.18632/oncotarget.9883.
Li, X., Zheng, L., Zhang, F., Hu, J., Chou, J., Liu, Y., et al. (2016c). STARD13-correlated ceRNA network inhibits EMT and metastasis of breast cancer. *Oncotarget* 7(17), 23197-23211. doi: 10.18632/oncotarget.8099.
Li, Y., Chen, D., Gao, X., Li, X., and Shi, G. (2017h). LncRNA NEAT1 Regulates Cell Viability and Invasion in Esophageal Squamous Cell Carcinoma through the miR-129/CTBP2 Axis. *Dis Markers* 2017, 5314649. doi: 10.1155/2017/5314649.
Li, Y., Lv, M., Song, Z., Lou, Z., Wang, R., and Zhuang, M. (2018d). Long non-coding RNA NNT-AS1 affects progression of breast cancer through miR-142-3p/ZEB1 axis. *Biomed Pharmacother* 103, 939-946. doi: 10.1016/j.biopha.2018.04.087.
Liang, C., Yang, Y., Guan, J., Lv, T., Qu, S., Fu, Q., et al. (2018). LncRNA UCA1 sponges miR-204-5p to promote migration, invasion and epithelial-mesenchymal transition of glioma cells via upregulation of ZEB1. *Pathol Res Pract* 214(9), 1474-1481. doi: 10.1016/j.prp.2018.07.036.
Liang, W.C., Fu, W.M., Wong, C.W., Wang, Y., Wang, W.M., Hu, G.X., et al. (2015). The lncRNA H19 promotes epithelial to mesenchymal transition by functioning as miRNA sponges in colorectal cancer. *Oncotarget* 6(26), 22513-22525. doi: 10.18632/oncotarget.4154.
Lin, J., Shi, Z., Yu, Z., and He, Z. (2018). LncRNA HIF1A-AS2 positively affects the progression and EMT formation of colorectal cancer through regulating miR-129-5p and DNMT3A. *Biomed Pharmacother* 98, 433-439. doi: 10.1016/j.biopha.2017.12.058.
Lin, Z., Ge, J., Wang, Z., Ren, J., Wang, X., Xiong, H., et al. (2017). Let-7e modulates the inflammatory response in vascular endothelial cells through ceRNA crosstalk. *Sci Rep* 7, 42498. doi: 10.1038/srep42498.

Liu, D., Zhu, Y., Pang, J., Weng, X., Feng, X., and Guo, Y. (2018a). Knockdown of long non-coding RNA MALAT1 inhibits growth and motility of human hepatoma cells via modulation of miR-195. *J Cell Biochem* 119(2)**,** 1368-1380. doi: 10.1002/jcb.26297.
Liu, H., Deng, H., Zhao, Y., Li, C., and Liang, Y. (2018b). LncRNA XIST/miR-34a axis modulates the cell proliferation and tumor growth of thyroid cancer through MET-PI3K-AKT signaling. *J Exp Clin Cancer Res* 37(1)**,** 279. doi: 10.1186/s13046-018-0950-9.
Liu, K., Yao, H., Wen, Y., Zhao, H., Zhou, N., Lei, S., et al. (2018c). Functional role of a long non-coding RNA LIFR-AS1/miR-29a/TNFAIP3 axis in colorectal cancer resistance to pohotodynamic therapy. *Biochim Biophys Acta Mol Basis Dis* 1864(9 Pt B)**,** 2871-2880. doi: 10.1016/j.bbadis.2018.05.020.
Liu, L., Yang, J., Zhu, X., Li, D., Lv, Z., and Zhang, X. (2016a). Long noncoding RNA H19 competitively binds miR-17-5p to regulate YES1 expression in thyroid cancer. *FEBS J* 283(12)**,** 2326-2339. doi: 10.1111/febs.13741.
Liu, M.L., Zhang, Q., Yuan, X., Jin, L., Wang, L.L., Fang, T.T., et al. (2018d). Long noncoding RNA RP4 functions as a competing endogenous RNA through miR-7-5p sponge activity in colorectal cancer. *World J Gastroenterol* 24(9)**,** 1004-1012. doi: 10.3748/wjg.v24.i9.1004.
Liu, Q., Guo, X., Que, S., Yang, X., Fan, H., Liu, M., et al. (2017a). LncRNA RSU1P2 contributes to tumorigenesis by acting as a ceRNA against let-7a in cervical cancer cells. *Oncotarget* 8(27)**,** 43768-43781. doi: 10.18632/oncotarget.10844.
Liu, Q., Li, Y., Lv, W., Zhang, G., Tian, X., Li, X., et al. (2018e). UCA1 promotes cell proliferation and invasion and inhibits apoptosis through regulation of the miR129-SOX4 pathway in renal cell carcinoma. *Onco Targets Ther* 11**,** 2475-2487. doi: 10.2147/OTT.S160192.
Liu, T., Chi, H., Chen, J., Chen, C., Huang, Y., Xi, H., et al. (2017b). Curcumin suppresses proliferation and in vitro invasion of human prostate cancer stem cells by ceRNA effect of miR-145 and lncRNA-ROR. *Gene* 631**,** 29-38. doi: 10.1016/j.gene.2017.08.008.
Liu, X., Ma, J., Xu, F., and Li, L. (2018f). TINCR suppresses proliferation and invasion through regulating miR-544a/FBXW7 axis in lung cancer. *Biomed Pharmacother* 99**,** 9-17. doi: 10.1016/j.biopha.2018.01.049.
Liu, X.H., Sun, M., Nie, F.Q., Ge, Y.B., Zhang, E.B., Yin, D.D., et al. (2014). Lnc RNA HOTAIR functions as a competing endogenous RNA to regulate HER2 expression by sponging miR-331-3p in gastric cancer. *Mol Cancer* 13**,** 92. doi: 10.1186/1476-4598-13-92.
Liu, Y., Tao, Z., Qu, J., Zhou, X., and Zhang, C. (2017c). Long non-coding RNA PCAT7 regulates ELF2 signaling through inhibition of miR-134-5p in nasopharyngeal carcinoma. *Biochem Biophys Res Commun* 491(2)**,** 374-381. doi: 10.1016/j.bbrc.2017.07.093.
Liu, Y., Wang, Y., Fu, X., and Lu, Z. (2018g). Long non-coding RNA NEAT1 promoted ovarian cancer cells' metastasis through regulation of miR-382-3p/ROCK1 axial. *Cancer Sci* 109(7)**,** 2188-2198. doi: 10.1111/cas.13647.
Liu, Z., Wei, X., Zhang, A., Li, C., Bai, J., and Dong, J. (2016b). Long non-coding RNA HNF1A-AS1 functioned as an oncogene and autophagy promoter in hepatocellular carcinoma through sponging hsa-miR-30b-5p. *Biochem Biophys Res Commun* 473(4)**,** 1268-1275. doi: 10.1016/j.bbrc.2016.04.054.
Lu, H., He, Y., Lin, L., Qi, Z., Ma, L., Li, L., et al. (2016a). Long non-coding RNA MALAT1 modulates radiosensitivity of HR-HPV+ cervical cancer via sponging miR-145. *Tumour Biol* 37(2)**,** 1683-1691. doi: 10.1007/s13277-015-3946-5.
Lu, M.H., Tang, B., Zeng, S., Hu, C.J., Xie, R., Wu, Y.Y., et al. (2016b). Long noncoding RNA BC032469, a novel competing endogenous RNA, upregulates hTERT expression by sponging miR-1207-5p and promotes proliferation in gastric cancer. *Oncogene* 35(27)**,** 3524-3534. doi: 10.1038/onc.2015.413.

Luan, W., Li, L., Shi, Y., Bu, X., Xia, Y., Wang, J., et al. (2016). Long non-coding RNA MALAT1 acts as a competing endogenous RNA to promote malignant melanoma growth and metastasis by sponging miR-22. *Oncotarget* 7(39), 63901-63912. doi: 10.18632/oncotarget.11564.

Luan, X., and Wang, Y. (2018). LncRNA XLOC_006390 facilitates cervical cancer tumorigenesis and metastasis as a ceRNA against miR-331-3p and miR-338-3p. *J Gynecol Oncol* 29(6), e95. doi: 10.3802/jgo.2018.29.e95.

Lv, J., Fan, H.X., Zhao, X.P., Lv, P., Fan, J.Y., Zhang, Y., et al. (2016). Long non-coding RNA Unigene56159 promotes epithelial-mesenchymal transition by acting as a ceRNA of miR-140-5p in hepatocellular carcinoma cells. *Cancer Lett* 382(2), 166-175. doi: 10.1016/j.canlet.2016.08.029.

Lv, L., Jia, J.Q., and Chen, J. (2018). The lncRNA CCAT1 Upregulates Proliferation and Invasion in Melanoma Cells via Suppressing miR-33a. *Oncol Res* 26(2), 201-208. doi: 10.3727/096504017X14920318811749.

Lv, M., Zhong, Z., Huang, M., Tian, Q., Jiang, R., and Chen, J. (2017). lncRNA H19 regulates epithelial-mesenchymal transition and metastasis of bladder cancer by miR-29b-3p as competing endogenous RNA. *Biochim Biophys Acta Mol Cell Res* 1864(10), 1887-1899. doi: 10.1016/j.bbamcr.2017.08.001.

Ma, C.C., Xiong, Z., Zhu, G.N., Wang, C., Zong, G., Wang, H.L., et al. (2016). Long non-coding RNA ATB promotes glioma malignancy by negatively regulating miR-200a. *J Exp Clin Cancer Res* 35(1), 90. doi: 10.1186/s13046-016-0367-2.

Ma, J., Fan, Y., Feng, T., Chen, F., Xu, Z., Li, S., et al. (2017). HOTAIR regulates HK2 expression by binding endogenous miR-125 and miR-143 in oesophageal squamous cell carcinoma progression. *Oncotarget* 8(49), 86410-86422. doi: 10.18632/oncotarget.21195.

Ma, J., Yang, Y., Huo, D., Wang, Z., Zhai, X., Chen, J., et al. (2018). LincRNA-RoR/miR-145 promote invasion and metastasis in triple-negative breast cancer via targeting MUC1. *Biochem Biophys Res Commun* 500(3), 614-620. doi: 10.1016/j.bbrc.2018.04.119.

Maldotti, M., Incarnato, D., Neri, F., Krepelova, A., Rapelli, S., Anselmi, F., et al. (2016). The long intergenic non-coding RNA CCR492 functions as a let-7 competitive endogenous RNA to regulate c-Myc expression. *Biochim Biophys Acta* 1859(10), 1322-1332. doi: 10.1016/j.bbagrm.2016.06.010.

Meng, Y.B., He, X., Huang, Y.F., Wu, Q.N., Zhou, Y.C., and Hao, D.J. (2017). Long Noncoding RNA CRNDE Promotes Multiple Myeloma Cell Growth by Suppressing miR-451. *Oncol Res* 25(7), 1207-1214. doi: 10.3727/096504017X14886679715637.

Mou, K., Liu, B., Ding, M., Mu, X., Han, D., Zhou, Y., et al. (2018). lncRNA-ATB functions as a competing endogenous RNA to promote YAP1 by sponging miR-590-5p in malignant melanoma. *Int J Oncol* 53(3), 1094-1104. doi: 10.3892/ijo.2018.4454.

Nie, W., Ge, H.J., Yang, X.Q., Sun, X., Huang, H., Tao, X., et al. (2016). LncRNA-UCA1 exerts oncogenic functions in non-small cell lung cancer by targeting miR-193a-3p. *Cancer Lett* 371(1), 99-106. doi: 10.1016/j.canlet.2015.11.024.

Pan, J., Li, X., Wu, W., Xue, M., Hou, H., Zhai, W., et al. (2016). Long non-coding RNA UCA1 promotes cisplatin/gemcitabine resistance through CREB modulating miR-196a-5p in bladder cancer cells. *Cancer Lett* 382(1), 64-76. doi: 10.1016/j.canlet.2016.08.015.

Peng, W., Si, S., Zhang, Q., Li, C., Zhao, F., Wang, F., et al. (2015). Long non-coding RNA MEG3 functions as a competing endogenous RNA to regulate gastric cancer progression. *J Exp Clin Cancer Res* 34, 79. doi: 10.1186/s13046-015-0197-7.

Pilyugin, M., and Irminger-Finger, I. (2014). Long non-coding RNA and microRNAs might act in regulating the expression of BARD1 mRNAs. *Int J Biochem Cell Biol* 54, 356-367. doi: 10.1016/j.biocel.2014.06.018.

Qin, N., Tong, G.F., Sun, L.W., and Xu, X.L. (2017). Long Noncoding RNA MEG3 Suppresses Glioma Cell Proliferation, Migration, and Invasion by Acting as a Competing Endogenous RNA of miR-19a. *Oncol Res* 25(9)**,** 1471-1478. doi: 10.3727/096504017X14886689179993.

Qu, Y., Xiao, H., Xiao, W., Xiong, Z., Hu, W., Gao, Y., et al. (2018). Upregulation of MIAT Regulates LOXL2 Expression by Competitively Binding MiR-29c in Clear Cell Renal Cell Carcinoma. *Cell Physiol Biochem* 48(3)**,** 1075-1087. doi: 10.1159/000491974.

Shang, M., Wang, X., Zhang, Y., Gao, Z., Wang, T., and Liu, R. (2018). LincRNA-ROR promotes metastasis and invasion of esophageal squamous cell carcinoma by regulating miR-145/FSCN1. *Onco Targets Ther* 11**,** 639-649. doi: 10.2147/OTT.S157638.

She, K., Yan, H., Huang, J., Zhou, H., and He, J. (2018). miR-193b availability is antagonized by LncRNA-SNHG7 for FAIM2-induced tumour progression in non-small cell lung cancer. *Cell Prolif* 51(1). doi: 10.1111/cpr.12406.

Shen, F., Chang, H., Gao, G., Zhang, B., Li, X., and Jin, B. (2018a). Long noncoding RNA FOXD2-AS1 promotes glioma malignancy and tumorigenesis via targeting miR-185-5p/CCND2 axis. *J Cell Biochem*. doi: 10.1002/jcb.28208.

Shen, Q., and Jiang, Y. (2018). LncRNA NNT-AS1 promotes the proliferation, and invasion of lung cancer cells via regulating miR-129-5p expression. *Biomed Pharmacother* 105**,** 176-181. doi: 10.1016/j.biopha.2018.05.123.

Shen, Y., Gao, X., Tan, W., and Xu, T. (2018b). STAT1-mediated upregulation of lncRNA LINC00174 functions a ceRNA for miR-1910-3p to facilitate colorectal carcinoma progression through regulation of TAZ. *Gene* 666**,** 64-71. doi: 10.1016/j.gene.2018.05.001.

Song, Y.X., Sun, J.X., Zhao, J.H., Yang, Y.C., Shi, J.X., Wu, Z.H., et al. (2017). Non-coding RNAs participate in the regulatory network of CLDN4 via ceRNA mediated miRNA evasion. *Nat Commun* 8(1)**,** 289. doi: 10.1038/s41467-017-00304-1.

Sui, J., Yang, X., Qi, W., Guo, K., Gao, Z., Wang, L., et al. (2017). Long Non-Coding RNA Linc-USP16 Functions As a Tumour Suppressor in Hepatocellular Carcinoma by Regulating PTEN Expression. *Cell Physiol Biochem* 44(3)**,** 1188-1198. doi: 10.1159/000485449.

Sun, C., Li, S., Zhang, F., Xi, Y., Wang, L., Bi, Y., et al. (2016). Long non-coding RNA NEAT1 promotes non-small cell lung cancer progression through regulation of miR-377-3p-E2F3 pathway. *Oncotarget* 7(32)**,** 51784-51814. doi: 10.18632/oncotarget.10108.

Sun, J.Y., Zhao, Z.W., Li, W.M., Yang, G., Jing, P.Y., Li, P., et al. (2017a). Knockdown of MALAT1 expression inhibits HUVEC proliferation by upregulation of miR-320a and downregulation of FOXM1 expression. *Oncotarget* 8(37)**,** 61499-61509. doi: 10.18632/oncotarget.18507.

Sun, Y., Cheng, H., Wang, G., Yu, G., Zhang, D., Wang, Y., et al. (2017b). Deregulation of miR-183 promotes melanoma development via lncRNA MALAT1 regulation and ITGB1 signal activation. *Oncotarget* 8(2)**,** 3509-3518. doi: 10.18632/oncotarget.13862.

Tan, H., and Zhao, L. (2018). lncRNA nuclear-enriched abundant transcript 1 promotes cell proliferation and invasion by targeting miR-186-5p/HIF-1alpha in osteosarcoma. *J Cell Biochem*. doi: 10.1002/jcb.27941.

Tan, H.Y., Wang, C., Liu, G., and Zhou, X. (2018). Long noncoding RNA NEAT1-modualted miR-506 regulates gastric cancer development through targeting STAT3. *J Cell Biochem*. doi: 10.1002/jcb.26691.

Tang, J., Zhuo, H., Zhang, X., Jiang, R., Ji, J., Deng, L., et al. (2014). A novel biomarker Linc00974 interacting with KRT19 promotes proliferation and metastasis in hepatocellular carcinoma. *Cell Death Dis* 5**,** e1549. doi: 10.1038/cddis.2014.518.

Tao, F., Tian, X., Ruan, S., Shen, M., and Zhang, Z. (2018). miR-211 sponges lncRNA MALAT1 to suppress tumor growth and progression through inhibiting PHF19 in ovarian carcinoma. *FASEB J***,** fj201800495RR. doi: 10.1096/fj.201800495RR.

Tu, J., Zhao, Z., Xu, M., Lu, X., Chang, L., and Ji, J. (2018). NEAT1 upregulates TGF-beta1 to induce hepatocellular carcinoma progression by sponging hsa-mir-139-5p. *J Cell Physiol* 233(11), 8578-8587. doi: 10.1002/jcp.26524.

Wang, C., Han, C., Zhang, Y., and Liu, F. (2018a). LncRNA PVT1 regulate expression of HIF1alpha via functioning as ceRNA for miR199a5p in nonsmall cell lung cancer under hypoxia. *Mol Med Rep* 17(1), 1105-1110. doi: 10.3892/mmr.2017.7962.

Wang, H., Huo, X., Yang, X.R., He, J., Cheng, L., Wang, N., et al. (2017a). STAT3-mediated upregulation of lncRNA HOXD-AS1 as a ceRNA facilitates liver cancer metastasis by regulating SOX4. *Mol Cancer* 16(1), 136. doi: 10.1186/s12943-017-0680-1.

Wang, J., Cao, L., Wu, J., and Wang, Q. (2018b). Long non-coding RNA SNHG1 regulates NOB1 expression by sponging miR-326 and promotes tumorigenesis in osteosarcoma. *Int J Oncol* 52(1), 77-88. doi: 10.3892/ijo.2017.4187.

Wang, O., Huang, Y., Wu, H., Zheng, B., Lin, J., and Jin, P. (2018c). LncRNA LOC728196/miR-513c axis facilitates glioma carcinogenesis by targeting TCF7. *Gene* 679, 119-125. doi: 10.1016/j.gene.2018.08.081.

Wang, S.H., Ma, F., Tang, Z.H., Wu, X.C., Cai, Q., Zhang, M.D., et al. (2016a). Long non-coding RNA H19 regulates FOXM1 expression by competitively binding endogenous miR-342-3p in gallbladder cancer. *J Exp Clin Cancer Res* 35(1), 160. doi: 10.1186/s13046-016-0436-6.

Wang, S.H., Zhang, W.J., Wu, X.C., Weng, M.Z., Zhang, M.D., Cai, Q., et al. (2016b). The lncRNA MALAT1 functions as a competing endogenous RNA to regulate MCL-1 expression by sponging miR-363-3p in gallbladder cancer. *J Cell Mol Med* 20(12), 2299-2308. doi: 10.1111/jcmm.12920.

Wang, S.H., Zhang, W.J., Wu, X.C., Zhang, M.D., Weng, M.Z., Zhou, D., et al. (2016c). Long non-coding RNA Malat1 promotes gallbladder cancer development by acting as a molecular sponge to regulate miR-206. *Oncotarget* 7(25), 37857-37867. doi: 10.18632/oncotarget.9347.

Wang, W.T., Ye, H., Wei, P.P., Han, B.W., He, B., Chen, Z.H., et al. (2016d). LncRNAs H19 and HULC, activated by oxidative stress, promote cell migration and invasion in cholangiocarcinoma through a ceRNA manner. *J Hematol Oncol* 9(1), 117. doi: 10.1186/s13045-016-0348-0.

Wang, Y., Liu, Z., Yao, B., Li, Q., Wang, L., Wang, C., et al. (2017b). Long non-coding RNA CASC2 suppresses epithelial-mesenchymal transition of hepatocellular carcinoma cells through CASC2/miR-367/FBXW7 axis. *Mol Cancer* 16(1), 123. doi: 10.1186/s12943-017-0702-z.

Wang, Y., Zeng, X., Wang, N., Zhao, W., Zhang, X., Teng, S., et al. (2018d). Long noncoding RNA DANCR, working as a competitive endogenous RNA, promotes ROCK1-mediated proliferation and metastasis via decoying of miR-335-5p and miR-1972 in osteosarcoma. *Mol Cancer* 17(1), 89. doi: 10.1186/s12943-018-0837-6.

Wu, F., Mo, Q., Wan, X., Dan, J., and Hu, H. (2017a). NEAT1/hsa-mir-98-5p/MAPK6 axis is involved in non-small-cell lung cancer development. *J Cell Biochem*. doi: 10.1002/jcb.26442.

Wu, H., and Zhou, C. (2018). Long non-coding RNA UCA1 promotes lung cancer cell proliferation and migration via microRNA-193a/HMGB1 axis. *Biochem Biophys Res Commun* 496(2), 738-745. doi: 10.1016/j.bbrc.2018.01.097.

Wu, X.S., Wang, F., Li, H.F., Hu, Y.P., Jiang, L., Zhang, F., et al. (2017b). LncRNA-PAGBC acts as a microRNA sponge and promotes gallbladder tumorigenesis. *EMBO Rep* 18(10), 1837-1853. doi: 10.15252/embr.201744147.

Xia, F., Chen, Y., Jiang, B., Du, X., Peng, Y., Wang, W., et al. (2018). Long Noncoding RNA HOXA-AS2 Promotes Papillary Thyroid Cancer Progression by Regulating miR-520c-3p/S100A4 Pathway. *Cell Physiol Biochem* 50(5), 1659-1672. doi: 10.1159/000494786.

Xia, T., Liao, Q., Jiang, X., Shao, Y., Xiao, B., Xi, Y., et al. (2014). Long noncoding RNA associated-competing endogenous RNAs in gastric cancer. *Sci Rep* 4, 6088. doi: 10.1038/srep06088.

Xiao, S., Wang, R., Wu, X., Liu, W., and Ma, S. (2018). The Long Noncoding RNA TP73-AS1 Interacted with miR-124 to Modulate Glioma Growth by Targeting Inhibitor of Apoptosis-Stimulating Protein of p53. *DNA Cell Biol* 37(2), 117-125. doi: 10.1089/dna.2017.3941.

Xiao, Y., Jiao, C., Lin, Y., Chen, M., Zhang, J., Wang, J., et al. (2017). lncRNA UCA1 Contributes to Imatinib Resistance by Acting as a ceRNA Against miR-16 in Chronic Myeloid Leukemia Cells. *DNA Cell Biol* 36(1), 18-25. doi: 10.1089/dna.2016.3533.

Xie, C., Chen, B., Wu, B., Guo, J., and Cao, Y. (2018a). LncRNA TUG1 promotes cell proliferation and suppresses apoptosis in osteosarcoma by regulating miR-212-3p/FOXA1 axis. *Biomed Pharmacother* 97, 1645-1653. doi: 10.1016/j.biopha.2017.12.004.

Xie, C.R., Wang, F., Zhang, S., Wang, F.Q., Zheng, S., Li, Z., et al. (2017). Long Noncoding RNA HCAL Facilitates the Growth and Metastasis of Hepatocellular Carcinoma by Acting as a ceRNA of LAPTM4B. *Mol Ther Nucleic Acids* 9, 440-451. doi: 10.1016/j.omtn.2017.10.018.

Xie, C.H., Cao, Y.M., Huang, Y., Shi, Q.W., Guo, J.H., Fan, Z.W., et al. (2016). Long non-coding RNA TUG1 contributes to tumorigenesis of human osteosarcoma by sponging miR-9-5p and regulating POU2F1 expression. *Tumour Biol* 37(11), 15031-15041. doi: 10.1007/s13277-016-5391-5.

Xie, Q., Lin, S., Zheng, M., Cai, Q., and Tu, Y. (2018b). Long noncoding RNA NEAT1 promoted the growth of cervical cancer cells via sponging miR-9-5p. *Biochem Cell Biol*. doi: 10.1139/bcb-2018-0111.

Xiong, D.D., Li, Z.Y., Liang, L., He, R.Q., Ma, F.C., Luo, D.Z., et al. (2018). The LncRNA NEAT1 Accelerates Lung Adenocarcinoma Deterioration and Binds to Mir-193a-3p as a Competitive Endogenous RNA. *Cell Physiol Biochem* 48(3), 905-918. doi: 10.1159/000491958.

Xu, C., He, T., Li, Z., Liu, H., and Ding, B. (2017a). Regulation of HOXA11-AS/miR-214-3p/EZH2 axis on the growth, migration and invasion of glioma cells. *Biomed Pharmacother* 95, 1504-1513. doi: 10.1016/j.biopha.2017.08.097.

Xu, J., Zhang, R., and Zhao, J. (2017b). The Novel Long Noncoding RNA TUSC7 Inhibits Proliferation by Sponging MiR-211 in Colorectal Cancer. *Cell Physiol Biochem* 41(2), 635-644. doi: 10.1159/000457938.

Xu, Y., Zhang, G., Zou, C., Zhang, H., Gong, Z., Wang, W., et al. (2018). LncRNA MT1JP Suppresses Gastric Cancer Cell Proliferation and Migration Through MT1JP/MiR-214-3p/RUNX3 Axis. *Cell Physiol Biochem* 46(6), 2445-2459. doi: 10.1159/000489651.

Xue, M., Pang, H., Li, X., Li, H., Pan, J., and Chen, W. (2016). Long non-coding RNA urothelial cancer-associated 1 promotes bladder cancer cell migration and invasion by way of the hsa-miR-145-ZEB1/2-FSCN1 pathway. *Cancer Sci* 107(1), 18-27. doi: 10.1111/cas.12844.

Xue, Y., Ni, T., Jiang, Y., and Li, Y. (2017). Long Noncoding RNA GAS5 Inhibits Tumorigenesis and Enhances Radiosensitivity by Suppressing miR-135b Expression in Non-Small Cell Lung Cancer. *Oncol Res* 25(8), 1305-1316. doi: 10.3727/096504017X14850182723737.

Yan, Y., Fan, Q., Wang, L., Zhou, Y., Li, J., and Zhou, K. (2017). LncRNA Snhg1, a non-degradable sponge for miR-338, promotes expression of proto-oncogene CST3 in primary esophageal cancer cells. *Oncotarget* 8(22), 35750-35760. doi: 10.18632/oncotarget.16189.

Yan, Z.Y., and Sun, X.C. (2018). [LincRNA-ROR functions as a ceRNA to regulate Oct4, Sox2, and Nanog expression by sponging miR-145 and its effect on biologic characteristics of colonic cancer stem cells]. *Zhonghua Bing Li Xue Za Zhi* 47(4), 284-290. doi: 10.3760/cma.j.issn.0529-5807.2018.04.011.

Yang, B.Y., Meng, Q., Sun, Y., Gao, L., and Yang, J.X. (2018a). Long non-coding RNA SNHG16 contributes to glioma malignancy by competitively binding miR-20a-5p with E2F1. *J Biol Regul Homeost Agents* 32(2), 251-261.

Yang, R., Li, P., Zhang, G., Lu, C., Wang, H., and Zhao, G. (2017). Long Non-Coding RNA XLOC_008466 Functions as an Oncogene in Human Non-Small Cell Lung Cancer by Targeting miR-874. *Cell Physiol Biochem* 42(1), 126-136. doi: 10.1159/000477121.

Yang, T., He, X., Chen, A., Tan, K., and Du, X. (2018b). LncRNA HOTAIR contributes to the malignancy of hepatocellular carcinoma by enhancing epithelial-mesenchymal transition via sponging miR-23b-3p from ZEB1. *Gene* 670, 114-122. doi: 10.1016/j.gene.2018.05.061.

Yang, T., Zhai, H., Yan, R., Zhou, Z., Gao, L., and Wang, L. (2018c). lncRNA CCAT1 promotes cell proliferation, migration, and invasion by down-regulation of miR-143 in FTC-133 thyroid carcinoma cell line. *Braz J Med Biol Res* 51(6), e7046. doi: 10.1590/1414-431x20187046.

Yao, J., Xu, F., Zhang, D., Yi, W., Chen, X., Chen, G., et al. (2018). TP73-AS1 promotes breast cancer cell proliferation through miR-200a-mediated TFAM inhibition. *J Cell Biochem* 119(1), 680-690. doi: 10.1002/jcb.26231.

Ye, Y., Gu, B., Wang, Y., Shen, S., and Huang, W. (2018). E2F1-mediated MNX1-AS1-miR-218-5p-SEC61A1 feedback loop contributes to the progression of colon adenocarcinoma. *J Cell Biochem*. doi: 10.1002/jcb.27902.

Yue, B., Sun, B., Liu, C., Zhao, S., Zhang, D., Yu, F., et al. (2015). Long non-coding RNA Fer-1-like protein 4 suppresses oncogenesis and exhibits prognostic value by associating with miR-106a-5p in colon cancer. *Cancer Sci* 106(10), 1323-1332. doi: 10.1111/cas.12759.

Zeng, H., Wang, J., Chen, T., Zhang, K., Chen, J., Wang, L., et al. (2018). Downregulation of long non-coding RNA Opa interacting protein 5-antisense RNA 1 inhibits breast cancer progression by targeting sex-determining region Y-box 2 by microRNA-129-5p upregulation. *Cancer Sci*. doi: 10.1111/cas.13879.

Zhan, Y., Chen, Z., Li, Y., He, A., He, S., Gong, Y., et al. (2018). Long non-coding RNA DANCR promotes malignant phenotypes of bladder cancer cells by modulating the miR-149/MSI2 axis as a ceRNA. *J Exp Clin Cancer Res* 37(1), 273. doi: 10.1186/s13046-018-0921-1.

Zhang, C.Z. (2017a). Long intergenic non-coding RNA 668 regulates VEGFA signaling through inhibition of miR-297 in oral squamous cell carcinoma. *Biochem Biophys Res Commun* 489(4), 404-412. doi: 10.1016/j.bbrc.2017.05.155.

Zhang, C.Z. (2017b). Long non-coding RNA FTH1P3 facilitates oral squamous cell carcinoma progression by acting as a molecular sponge of miR-224-5p to modulate fizzled 5 expression. *Gene* 607, 47-55. doi: 10.1016/j.gene.2017.01.009.

Zhang, D., Cao, J., Zhong, Q., Zeng, L., Cai, C., Lei, L., et al. (2017a). Long noncoding RNA PCAT-1 promotes invasion and metastasis via the miR-129-5p-HMGB1 signaling pathway in hepatocellular carcinoma. *Biomed Pharmacother* 95, 1187-1193. doi: 10.1016/j.biopha.2017.09.045.

Zhang, L., Fang, F., and He, X. (2018a). Long noncoding RNA TP73-AS1 promotes non-small cell lung cancer progression by competitively sponging miR-449a/EZH2. *Biomed Pharmacother* 104, 705-711. doi: 10.1016/j.biopha.2018.05.089.

Zhang, L., Lu, X.Q., Zhou, X.Q., Liu, Q.B., Chen, L., and Cai, F. (2019). NEAT1 induces osteosarcoma development by modulating the miR-339-5p/TGF-beta1 pathway. *J Cell Physiol* 234(4), 5097-5105. doi: 10.1002/jcp.27313.

Zhang, Q., Chen, B., Liu, P., and Yang, J. (2018b). XIST promotes gastric cancer (GC) progression through TGF-beta1 via targeting miR-185. *J Cell Biochem* 119(3), 2787-2796. doi: 10.1002/jcb.26447.

Zhang, R., Guo, Y., Ma, Z., Ma, G., Xue, Q., Li, F., et al. (2017b). Long non-coding RNA PTENP1 functions as a ceRNA to modulate PTEN level by decoying miR-106b and miR-93 in gastric cancer. *Oncotarget* 8(16), 26079-26089. doi: 10.18632/oncotarget.15317.

Zhang, R., Jin, H., and Lou, F. (2018c). The Long Non-Coding RNA TP73-AS1 Interacted With miR-142 to Modulate Brain Glioma Growth Through HMGB1/RAGE Pathway. *J Cell Biochem* 119(4), 3007-3016. doi: 10.1002/jcb.26021.

Zhang, R., and Xia, T. (2017). Long non-coding RNA XIST regulates PDCD4 expression by interacting with miR-21-5p and inhibits osteosarcoma cell growth and metastasis. *Int J Oncol* 51(5), 1460-1470. doi: 10.3892/ijo.2017.4127.

Zhang, S., Dong, X., Ji, T., Chen, G., and Shan, L. (2017c). Long non-coding RNA UCA1 promotes cell progression by acting as a competing endogenous RNA of ATF2 in prostate cancer. *Am J Transl Res* 9(2), 366-375.

Zhang, X.N., Zhou, J., and Lu, X.J. (2018d). The long noncoding RNA NEAT1 contributes to hepatocellular carcinoma development by sponging miR-485 and enhancing the expression of the STAT3. *J Cell Physiol* 233(9), 6733-6741. doi: 10.1002/jcp.26371.

Zhang, Y., Chen, Z., Li, M.J., Guo, H.Y., and Jing, N.C. (2017d). Long non-coding RNA metastasis-associated lung adenocarcinoma transcript 1 regulates the expression of Gli2 by miR-202 to strengthen gastric cancer progression. *Biomed Pharmacother* 85, 264-271. doi: 10.1016/j.biopha.2016.11.014.

Zhang, Y., Hu, J., Zhou, W., and Gao, H. (2018e). LncRNA FOXD2-AS1 accelerates the papillary thyroid cancer progression through regulating the miR-485-5p/KLK7 axis. *J Cell Biochem*. doi: 10.1002/jcb.28072.

Zhang, Y., Liu, Y., and Xu, X. (2018f). Knockdown of LncRNA-UCA1 suppresses chemoresistance of pediatric AML by inhibiting glycolysis through the microRNA-125a/hexokinase 2 pathway. *J Cell Biochem* 119(7), 6296-6308. doi: 10.1002/jcb.26899.

Zhang, Z., Cheng, J., Wu, Y., Qiu, J., Sun, Y., and Tong, X. (2016). LncRNA HOTAIR controls the expression of Rab22a by sponging miR-373 in ovarian cancer. *Mol Med Rep* 14(3), 2465-2472. doi: 10.3892/mmr.2016.5572.

Zhang, Z., Liu, F., Yang, F., and Liu, Y. (2018g). Kockdown of OIP5-AS1 expression inhibits proliferation, metastasis and EMT progress in hepatoblastoma cells through up-regulating miR-186a-5p and down-regulating ZEB1. *Biomed Pharmacother* 101, 14-23. doi: 10.1016/j.biopha.2018.02.026.

Zhao, L., Han, T., Li, Y., Sun, J., Zhang, S., Liu, Y., et al. (2017). The lncRNA SNHG5/miR-32 axis regulates gastric cancer cell proliferation and migration by targeting KLF4. *FASEB J* 31(3), 893-903. doi: 10.1096/fj.201600994R.

Zhao, X., Li, X., Zhou, L., Ni, J., Yan, W., Ma, R., et al. (2018). LncRNA HOXA11-AS drives cisplatin resistance of human LUAD cells via modulating miR-454-3p/Stat3. *Cancer Sci* 109(10), 3068-3079. doi: 10.1111/cas.13764.

Zhong, H., Yang, J., Zhang, B., Wang, X., Pei, L., Zhang, L., et al. (2018). LncRNA GACAT3 predicts poor prognosis and promotes cell proliferation in breast cancer through regulation of miR-497/CCND2. *Cancer Biomark* 22(4), 787-797. doi: 10.3233/CBM-181354.

Zhou, J., Liu, M., Chen, Y., Xu, S., Guo, Y., and Zhao, L. (2018a). Cucurbitacin B suppresses proliferation of pancreatic cancer cells by ceRNA: Effect of miR-146b-5p and lncRNA-AFAP1-AS1. *J Cell Physiol*. doi: 10.1002/jcp.27264.

Zhou, P., Sun, L., Liu, D., Liu, C., and Sun, L. (2016). Long Non-Coding RNA lincRNA-ROR Promotes the Progression of Colon Cancer and Holds Prognostic Value by Associating with miR-145. *Pathol Oncol Res* 22(4), 733-740. doi: 10.1007/s12253-016-0061-x.

Zhou, S., Yu, L., Xiong, M., and Dai, G. (2018b). LncRNA SNHG12 promotes tumorigenesis and metastasis in osteosarcoma by upregulating Notch2 by sponging miR-195-5p. *Biochem Biophys Res Commun* 495(2)**,** 1822-1832. doi: 10.1016/j.bbrc.2017.12.047.

Zhou, X., Gao, Q., Wang, J., Zhang, X., Liu, K., and Duan, Z. (2014). Linc-RNA-RoR acts as a "sponge" against mediation of the differentiation of endometrial cancer stem cells by microRNA-145. *Gynecol Oncol* 133(2)**,** 333-339. doi: 10.1016/j.ygyno.2014.02.033.

Zhu, H., Zheng, T., Yu, J., Zhou, L., and Wang, L. (2018a). LncRNA XIST accelerates cervical cancer progression via upregulating Fus through competitively binding with miR-200a. *Biomed Pharmacother* 105**,** 789-797. doi: 10.1016/j.biopha.2018.05.053.

Zhu, S.P., Wang, J.Y., Wang, X.G., and Zhao, J.P. (2017). Long intergenic non-protein coding RNA 00858 functions as a competing endogenous RNA for miR-422a to facilitate the cell growth in non-small cell lung cancer. *Aging (Albany NY)* 9(2)**,** 475-486. doi: 10.18632/aging.101171.

Zhu, Y., Qiao, L., Zhou, Y., Ma, N., Wang, C., and Zhou, J. (2018b). Long non-coding RNA FOXD2-AS1 contributes to colorectal cancer proliferation through its interaction with microRNA-185-5p. *Cancer Sci* 109(7)**,** 2235-2242. doi: 10.1111/cas.13632.

Zou, Q., Zhou, E., Xu, F., Zhang, D., Yi, W., and Yao, J. (2018). A TP73-AS1/miR-200a/ZEB1 regulating loop promotes breast cancer cell invasion and migration. *J Cell Biochem* 119(2)**,** 2189-2199. doi: 10.1002/jcb.26380.
